# Cholesterol and COVID-19—therapeutic opportunities at the host/virus interface during cell entry

**DOI:** 10.26508/lsa.202302453

**Published:** 2024-02-22

**Authors:** Thomas Grewal, Mai Khanh Linh Nguyen, Christa Buechler

**Affiliations:** 1 School of Pharmacy, Faculty of Medicine and Health, University of Sydney , Sydney, Australia; 2 Department of Internal Medicine I, Regensburg University Hospital , Regensburg, Germany

## Abstract

The rapid development of vaccines to combat severe acute respiratory syndrome coronavirus 2 (SARS-CoV-2) infections has been critical to reduce the severity of COVID-19. However, the continuous emergence of new SARS-CoV-2 subtypes highlights the need to develop additional approaches that oppose viral infections. Targeting host factors that support virus entry, replication, and propagation provide opportunities to lower SARS-CoV-2 infection rates and improve COVID-19 outcome. This includes cellular cholesterol, which is critical for viral spike proteins to capture the host machinery for SARS-CoV-2 cell entry. Once endocytosed, exit of SARS-CoV-2 from the late endosomal/lysosomal compartment occurs in a cholesterol-sensitive manner. In addition, effective release of new viral particles also requires cholesterol. Hence, cholesterol-lowering statins, proprotein convertase subtilisin/kexin type 9 antibodies, and ezetimibe have revealed potential to protect against COVID-19. In addition, pharmacological inhibition of cholesterol exiting late endosomes/lysosomes identified drug candidates, including antifungals, to block SARS-CoV-2 infection. This review describes the multiple roles of cholesterol at the cell surface and endolysosomes for SARS-CoV-2 entry and the potential of drugs targeting cholesterol homeostasis to reduce SARS-CoV-2 infectivity and COVID-19 disease severity.

## Abstract (precis)

The key to cholesterol-related broad-spectrum antiviral strategies may be to disrupt cholesterol handling in specific cellular organelles.

## Introduction

COVID-19 caused by infections with SARS-CoV-2 are accompanied by heterogeneous indications ranging from asymptomatic infections to serious and life-threatening manifestations, representing a major challenge to foretell COVID-19 disease outcome (Bean et al, 2023). Besides advanced age and male sex (Booth et al, 2021; Sieurin et al, 2022), other predictors for adverse outcomes in SARS-CoV-2–infected patients include obesity, cardiovascular disease, hypertension, type 2 diabetes, and other diseases such as cancer, kidney diseases, obstructive pulmonary disease, or pre-existing cerebrovascular and respiratory diseases (Booth et al, 2021; Cheng et al, 2021; Ng et al, 2021; Westheim et al, 2021).

### Targeting SARS-CoV-2 entry and trafficking along the endocytic pathway

Although the rapid development of vaccines greatly reduced the severity of COVID-19, the continuous emergence of new virus variants underscores antivirals to remain essential for the management of SARS-CoV-2 infections. Antivirals that target viral proteins include the clinically approved drug nirmatrelvir, which inhibits the viral major protease (M^pro^), and consequently, SARS-CoV-2 replication. Paxlovid combines nirmatrelvir with the HIV protease inhibitor ritonavir. The latter inhibits the cytochrome P450 3A4 enzyme, which increases the half-life of nirmatrelvir, providing antiviral activity against existing coronavirus variants (Blair, 2023).

Lopinavir is another potent HIV-1 protease inhibitor, leading to the production of immature, non-infectious virions. Although its combination with ritonavir increased bioavailability, this drug combination was associated with worse clinical outcomes in hospitalized COVID-19 patients (Babayigit et al, 2022).

Alternatively, the repurposing of drugs that target host factors hijacked by the virus shows promise. This includes the antiviral and immunomodulatory activities of the Food and Drug Administration (FDA)–approved antiparasitic ivermectin. Multiple drug–protein interactions with viral and host proteins at the cell surface and cytoplasm interfere with virus entry and propagation and provide anti-inflammatory activities targeting JAK/signal transducer and activator of transcription (STAT), phosphatidylinositol-3 kinase/protein kinase B (PI3K/Akt), and nuclear factor kappa-light-chain enhancer of activated B cells (NF-kB) signaling pathways. However, evidence of its therapeutic benefits to treat COVID-19 is still lacking (Shafiee et al, 2023).

Angiotensin-converting enzyme 2 (ACE2) is the major host cell receptor enabling SARS-CoV-2 to attach to the host cell surface. In the respiratory system, ACE2 was not detectable or expressed at low levels in some cells but was present in many other cells and tissues such as enterocytes, gallbladder, cardiomyocytes, ductal cells, and vasculature, indicating that high ACE2 expression is not an indicator of increased susceptibility to infection (Hikmet et al, 2020). Consistent with these findings, only a relatively small number of ACE2-positive cells were found in the human respiratory tract. Further indicating that ACE2 protein levels per se do not determine susceptibility to COVID-19 infection, increased ACE2 protein levels were found in individuals at lower risk of severe COVID-19, such as children and healthy controls (Ortiz et al, 2020).

Other host cell receptors for SARS-CoV-2 include neuropilin receptors, C-lectin–type receptors, cluster of differentiation 147 (CD147), heparan sulfate proteoglycans, dendritic cell-specific intercellular adhesion molecule-3-grabbing non-integrin (CD-SIGN), liver/lymph node-specific intercellular adhesion molecule-3-grabbing integrin (L-SIGN), macrophage galactose-type lectin, glucose-regulated protein 78, AXL receptor tyrosine kinase, and T-cell immunoglobulin and mucin domain protein 1 (reviewed in Cesar-Silva et al [2022]). The virus then enters the cell via fusion with the plasma membrane or clathrin-dependent and -independent endocytosis (Al-Horani et al, 2020; Sumbria et al, 2020). Current vaccines targeting the initial interaction of viral and host cell proteins at the cell surface have greatly contributed to combat COVID-19 disease.

**Figure 1.**
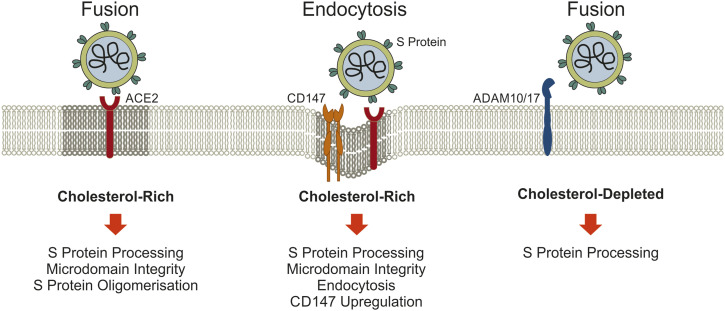
Cholesterol at the cell surface influences SARS-CoV-2 cell entry. (i) Cholesterol depletion displaces ACE2 from rafts and reduces SARS-CoV-2 docking and ACE2-dependent viral envelope fusion with the host membrane as well as endocytotic virus uptake. Spike protein oligomerization improves viral membrane fusion and is enhanced by cholesterol. (ii) Cholesterol loading of cells induces CD147 levels, which can mediate SARS-CoV-2 endocytosis and contribute to the higher risk for severe COVID-19 in dyslipidemia. (iii) ADAM10/17 contribute to S protein processing and are activated in cholesterol-depleted cells, a route that may overcome reduced infection efficacy when cell surface cholesterol levels are low. Abbreviations: ADAM 10/17, A Disintegrin And Metalloproteinase 10/17; ACE2, angiotensin-converting enzyme 2; CD147, cluster of differentiation 147; SARS-CoV-2, severe acute respiratory syndrome coronavirus 2; S protein, spike protein.

Internalized viruses trafficking along the endocytic pathway reach late endosomes/lysosomes (LE/Lys) and then exit this compartment to replicate and build new viral particles in the cytoplasm. Hence, more than 50 proteins and adaptors of the endocytic machinery showed therapeutic potential, with gene knockdown approaches of key players blocking SARS-CoV-2 entry (dynamin, clathrin, and caveolin) or small molecules such as dynasore (dynamin), pitstop 2 (clathrin-coated pit formation), chlorpromazine (clathrin-dependent endocytosis), rottlerin, amiloride (macropinocytosis), and LY294002 (phagocytosis) reducing SARS-CoV-2 infection (Bayati et al, 2021; Li et al, 2021b; Cesar-Silva et al, 2022; Alkafaas et al, 2023). Meplazumab, an antibody against the SARS-CoV-2 receptor CD147 (Fig 1), inhibited virus entry and amplification in fibroblasts, reducing the production of extracellular matrix proteins contributing to pulmonary fibrosis (Wang et al, 2020a; Wu et al, 2022). Dominant-negative mutants of Rab5 and Rab7 GTPases, which regulate the trafficking of vesicles along the endocytic pathway, also displayed an ability to block SARS-CoV-2 infection (Zang et al, 2020).

In addition, the targeting of cathepsins, endolysosomal cysteine proteases that are required for the processing of internalized virus to enter the cytoplasm, can lower SARS-CoV-2 infection. This includes cysteine protease inhibitors such as E64d and K11777 and cathepsin L–specific compounds Z-FY9t-Bu-DMK and teicoplanin (Huang et al, 2006; Zhou et al, 2015, 2016).

Furthermore, lysosomotropic agents, which cause lysosomal membrane permeabilization and dysfunction, have been considered to inhibit coronavirus infection This comprises hydroxychloroquine, which is used to treat malaria and several autoimmune diseases, and has antiviral effects due to its ability to compromise endo- and lysosomal acidification. Although large-scale randomized controlled trials failed to show any survival benefit of hydroxychloroquine for COVID-19 patients, it was approved by the FDA for emergency COVID-19 therapy (Self et al, 2020). Of note, in several clinical studies with >500 participants, twice or more the FDA-recommended doses of hydroxychloroquine were used, yet these studies did not provide conclusive evidence of the effectiveness of hydroxychloroquine in treating COVID-19 (Axfors et al, 2021; Kumar et al, 2021). Yet, hydroxychloroquine, as well as chloroquine, can severely compromise vital LE/Lys functions, and very high doses can lead to cell death and strong side effects and toxicity profiles.

Nevertheless, several acidification inhibitors block the exit of endocytosed SARS-CoV-2 from LE/Lys to significantly reduce infectivity of SARS-CoV-2 and other coronaviruses (Hoffmann et al, 2020; Prabhakara et al, 2021). However, the suitability of niclosamide, bafilomycin A1, and NH_4_Cl to treat SARS-CoV-2 infections requires further fine-tuning, as de-acidification results in profound changes and dysfunction of the LE/Lys compartment, many of those mandatory for fundamental cellular functions. Alternatively, inhibiting the maturation of LE to Lys, using apilimod and YM201636, effectively reduced SARS-CoV-2 infections (Kang et al, 2020). Hence, the host’s cell surface receptors and endocytic compartment provide a variety of therapeutic avenues to lower SARS-CoV-2 infection.

### Role of cholesterol at the plasma membrane for SARS-CoV-2 infection

Various human pathogenic viruses depend on the hosts’ lipid metabolism for infection and replication. Patients with severe infectious diseases have low levels of low-density lipoprotein (LDL) and high-density lipoprotein (HDL), and cholesterol loading of immune cells and most likely further cells and tissues seems to contribute to hypolipidemia (Wang et al, 2023). Accordingly, serum lipoprotein levels have prognostic value to predict the severity and mortality of patients with COVID-19 (Liu et al, 2021).

Cellular uptake of LDL- and HDL-derived cholesterol rapidly influences cholesterol levels at the plasma membrane and endocytic vesicles including LE/Lys, and dyslipidemia has been implicated to support SARS-CoV-2 infection by altering membrane lipid composition and interfering with immune cell function (Makhoul et al, 2022). Importantly, cell surface binding, cellular uptake, and propagation of SARS-CoV-2 are intimately linked to cellular cholesterol metabolism and distribution, providing therapeutic opportunities (Wang et al, 2022; Talukder et al, 2023).

The plasma membrane contains 60–80% of total cellular cholesterol, which represents 30–40% of all plasma membrane lipids. Moreover, cell surface cholesterol preferentially partitions in cholesterol-rich membrane domains (lipid rafts) containing receptors and functioning as signaling hubs to transmit environmental cues (Mesmin & Maxfield, 2009; Enrich et al, 2015). These specialized cholesterol-rich microdomains are heterogenous in terms of their lipid and protein content, often conferring different functions. For instance, lipid rafts enriched in either saturated ceramide-containing glycolipids such as monosialotetrahexosylganglioside 1 (GM1) or phosphatidyl-inositol 4,5 bisphosphate (PIP2) containing lipid clusters exist and movement of GM1-localized membrane proteins to PIP2 lipid clusters upon localized changes in membrane cholesterol levels may affect their biologic function (Yuan & Hansen, 2023). Lipid rafts also contain receptors that are recognized by viruses for cell entry, and disruption of lipid rafts using cholesterol-depleting agents such as methyl-β-cyclodextrin (MβCD), which can remove up to 80% of cellular cholesterol, strongly reduced the entry, infectivity, and release of murine coronavirus, SARS-CoV, and SARS-CoV-2 (Thorp & Gallagher, 2004; Li et al, 2007; Li et al, 2021b) (reviewed in Fecchi et al [2020]). ACE2 locates to lipid rafts and statin-induced cholesterol depletion reduced SARS-CoV-2 internalization in human epithelial lung adenocarcinoma Calu-3a cells (Cesar-Silva et al, 2022) (Fig 1). As ACE2 protein levels remained unchanged upon MβCD treatment, the association of ACE2 with cholesterol-rich membrane domains appears important for viral entry (Lu et al, 2008) (Fig 1). Interestingly, in a diabetic mouse model characterized by elevated cholesterol with increased age and disease, ACE2 was predominantly found in cholesterol-rich GM1 membrane structures that facilitate endocytosis and cholesterol depletion caused ACE2 to move from GM1 lipid to PIP2 lipid domains, thereby decreasing virus uptake and infectivity (Wang et al, 2023). Super-resolution microscopy also revealed a relationship between the spike protein and PIP2 clusters (Raut et al, 2022). In addition, hydroxychloroquine altered the association of ACE2 with GM1- and PIP2-containing microdomain clusters in cells with high and low cholesterol, respectively (Yuan et al, 2022). Also, avasimibe-mediated inhibition of acyl-CoA:cholesterol acyltransferase (ACAT)–mediated cholesterol esterification disrupted ACE2 association with GM1 lipid rafts and inhibited SARS-CoV-2 pseudoparticle infection (Wing et al, 2023).

Besides using the cholesterol-depleting agent MβCD, apolipoprotein E (ApoE) is an endogenous cholesterol transport protein that in the non-lipidated form removes cholesterol, whereas the lipidated form is a physiological cholesterol donor for cells. Most relevant for COVID-19, lipidated ApoE induced ACE2 movement to GM1 rafts and enhanced virus infection (Wang et al, 2023). Three apoE isoforms exist in humans—apoE2, apoE3, and apoE4—and it is in this order that these genotypes are associated with increasing LDL-cholesterol levels and coronary artery disease (Tudorache et al, 2017). Homozygous apoE4 carriers had an increased risk of severe COVID-19 infection, independent of preexisting comorbidities (Kuo et al, 2020). As the apoE4 genotype is associated with higher tissue cholesterol levels, oxidative stress, and inflammation, this may all contribute to greater susceptibility to SARS-CoV-2 infection and COVID-19 severity (Gkouskou et al, 2021).

Cholesterol-depleting cyclodextrins reduced binding of the S1 subunit of the viral spike (S) protein to ACE2 in Wuhan-Hu-1, Delta, and Omicron virus variants (Kovacs et al, 2023), which was reinforced by cholesterol depletion studies in SARS-CoV–infected Vero-E6 (African green monkey epithelial kidney cell line) and Calu-3a cells (Li et al, 2007; Lu et al, 2008; Wang et al, 2020b). These observations might relate to clinical settings, as pantethine, a vitamin B5 derivative, which reduces total and LDL-cholesterol, was associated with reduced SARS-CoV-2 infection (Abou-Hamdan et al, 2023).

Besides raft-dependent endocytosis, cholesterol can also support fusion of the viral envelope with raft domains at the plasma membrane (Ripa et al, 2021; Sanders et al, 2021; Cesar-Silva et al, 2022) and several cholesterol-sensitive players appear to be involved in the fusion process (Tang et al, 2021a). After docking of the S protein to ACE2, fusion of the host and viral membranes facilitate viral entry. This route depends on the processing of the viral S2 subunit by transmembrane serine protease type II (TMPRSS2) (Bestle et al, 2020).

Alternatively, fusion of endocytosed viruses with endosomal and cholesterol-rich membranes requires cathepsin L to cleave the S protein (Huang et al, 2006; Li et al, 2007; Zhou et al, 2015, 2016; Bestle et al, 2020; Yang & Shen, 2020). Besides furin and TMPRSS2 (Al-Horani et al, 2020; Nejat et al, 2023), a disintegrin and metalloproteinase (ADAM) 10 and 17 contribute to the processing of the viral S2 subunit (Jocher et al, 2022). Showing potential as therapeutic targets, ADAM protease inhibitors lowered SARS-CoV-2 infection in lung cells (Jocher et al, 2022) and the FDA-approved serine protease inhibitor clamostat blocked SARS-CoV-2 entry by inhibiting ACE2 and TMPRSS2 (Hoffmann et al, 2020). Platycodin D from *Platycodon grandifloras* redistributed membrane cholesterol, thereby antagonizing fusion of viral and cell membranes and preventing SARS-CoV-2 entry via endosomal and TMPRSS2 pathways (Kim et al, 2021b). Interestingly, cholesterol depletion enhanced ADAM10- and 17-mediated substrate cleavage (Reiss & Bhakdi, 2017), yet if this impacts on SARS-CoV-2 infection remains to be clarified (Fig 1).

Furthermore, membrane cholesterol accelerates the formation of oligomeric S protein structures that enhance S2 activity (Meher et al, 2023) (Fig 1). Host site-1 protease (S1P)–mediated activation of sterol regulatory element–binding protein 2 (SREBP2), the major transcription factor that controls cholesterol homeostatsis, increased SARS-CoV-2 fusion with the cell membrane due to enhanced S2 processing in a HeLa (human cervical carcinoma) cell model (Essalmani et al, 2023).

Of note, MβCD-sensitive entry mechanism mediated by the spike protein, yet independent of clathrin-coated pits and caveolae (Wang et al, 2008), further illustrate the diverse roles how cellular cholesterol at the cell surface can modulate SARS-CoV-2 cell entry (Wang et al, 2008; Essalmani et al, 2023).

Besides cholesterol-depleting agents, oxysterols such as 25-hydroxycholesterol can also reduce the availability of cell surface cholesterol, with inhibitory consequences for viral infectivity (reviewed in Mao et al [2022]). In Calu-3a cells, Caco-2 cells, and lung organoids, 25-hydroxycholesterol interfered with the fusion of SARS-CoV-2 with the cell surface (Wang et al, 2020b), most likely because of the transfer of cell surface cholesterol to lipid droplets. Alternatively, in HEK293 (human embryonic kidney) cells, 25-hydroxycholesterol accumulated in LE/Lys and blocked cholesterol export from this compartment, which appeared to inhibit SARS-CoV-2 spike protein-mediated fusion with the LE/Lys membrane (Zang et al, 2020). Notably, cholesterol-25-hydroxylase, which generates 25-hydroxycholesterol, is stimulated by interferon, up-regulated by SARS-CoV-2 infection and in COVID-19 patients, indicating that the generation of this oxysterol is part of the innate immune response to combat SARS-CoV-2 propagation (Mao et al, 2022).

### Cholesterol accumulation in LE/Lys and the role of Niemann–Pick type C1 protein for infection

As outlined above, disruption of cholesterol-sensitive mechanisms at the cell surface can interfere with SARS-CoV-2 cell entry (see also Fig 1). Yet, inhibition of protease TMPRSS2 at the plasma membrane in combination with blocked endolysosomal cathepsins completely abrogated SARS-CoV-2 entry (Hoffmann et al, 2020), identifying the endocytic pathway as another prominent route for SARS-CoV-2 cell entry (Ou et al, 2020; Tang et al, 2020), and a plethora of drugs that interfere with endocytic trafficking of SARS-CoV-2 have been described in the previous chapter above. Remarkably, more recent variants of SARS-CoV-2, such as the Omicron BA.1 variant, displayed a lower efficiency in using the cellular protease TMPRSS2 and relied more on cell entry through endocytic pathways that are sensitive to cholesterol (Meng et al, 2022). A recent study highlighting the requirement of an acidic endosomal environment for early variants of SARS-CoV-2 also point at the potential to target late endosomal cholesterol (Kreutzberger et al, 2022). Hence, drugs targeting endocytic pathways and late endosomal cholesterol may be effective for therapy of patients infected with both recent and earlier variants.

In fact, many zoonotically transmitted viruses, in particular enveloped viruses including SARS-CoV-2 (Tang et al, 2020), hijack late endosomal proteins to release their viral genome into the host cell. This includes the main cholesterol transporter in LE/Lys, Niemann–Pick type C1 (NPC1), which serves as the entry factor for several filoviruses with Ebola virus using NPC1 in a cholesterol-independent manner (Carette et al, 2011; Cote et al, 2011). In addition, cholesterol accumulation in LE/Lys, using the pharmacological NPC1 inhibitor U18666A, compromised fusion of the influenza lipid envelope with late endosomal membranes (Musiol et al, 2013; Kuhnl et al, 2018; Schloer et al, 2019). Alike, U18666A and other drugs that cause LE/Lys cholesterol accumulation elicit antiviral activity (Sturley et al, 2020) and specifically, U18666A reduced SARS-CoV-2 infection in Vero-E6 and Calu-3a cells (Schloer et al, 2020a). Thus, the disruption of cholesterol export from LE/Lys could serve as a pharmacological tool against SARS-CoV-2 (Schloer et al, 2020a; Sturley et al, 2020), but (Schloer et al, 2020a; Sturley et al, 2020) the cytotoxicity limits the therapeutical use of U18666A (Cenedella et al, 2004).

Alternatively, hydroxypropyl-beta-cyclodextrin serves as a cholesterol-depleting agent at the cell surface to reduce cholesterol accumulation in patients with NPC1 deficiency. This pharmaceutical impaired SARS-CoV-2 infection and replication and reduced expression of pro-inflammatory cytokines in human monocytes and Calu-3a cells (Bezerra et al, 2022). As hydroxypropyl-beta-cyclodextrin removes cholesterol from LE/Lys, the plasma membrane (Ottinger et al, 2014) and membranous organelles containing viral replication complexes (Bezerra et al, 2022), its mode of action to reduce SARS-CoV-2 infectivity remains to be clarified. Alike studies using MβCD (Lu et al, 2008), hydroxypropyl-beta-cyclodextrin did not alter ACE2 levels (Bezerra et al, 2022), indicating that these cholesterol-depleting cyclodextrins cause displacement of ACE2 from cholesterol-rich lipid rafts and consequently, reduced binding of the spike protein (Thorp & Gallagher, 2004; Li et al, 2007; Lu et al, 2008; Redondo-Morata et al, 2016; Kim et al, 2019; Fecchi et al, 2020; Cesar-Silva et al, 2022).

Miglustat inhibits glucosylceramide synthase, limiting ganglioside buildup in LE/Lys in several lysosomal storage disorders, such as Tay-Sachs and Sandhoff disease (Mansouri et al, 2023). In NPC patients, glycosphingolipid levels in LE/Lys are also increased and miglustat is approved to ameliorate neuronal dysfunction in NPC1 deficiency (Lachmann et al, 2004). As miglustat is an iminosugar, this compound also inhibits α-glucosidases I and II in the ER, responsible for early stages of glycoprotein N-linked oligosaccharide processing. The spike, envelope, and membrane proteins of SARS-CoV-2 are highly glycosylated proteins (Gong et al, 2021), and in SARS-CoV-2–infected cells, miglustat markedly decreased viral proteins, including the spike protein, and subsequent release of infectious viruses (Rajasekharan et al, 2021).

Other pharmacologicals with antiviral activities that cause LE/Lys-cholesterol accumulation include the antifungal itraconazole, a triazole derivative that blocks the synthesis of fungal ergosterol, but also binds and inhibits NPC1 (Trinh et al, 2017). Itraconazole has antiviral properties against a range of viruses in cell and mouse models (Schloer et al, 2019; Schloer et al, 2020b) and reduced SARS-CoV-2 infection in several cell lines (Liesenborghs et al, 2021; Schloer et al, 2021). However, a preclinical hamster model and a pilot clinical trial indicated this drug to lack clinical benefit (Liesenborghs et al, 2021).

In addition, inhibition of acid sphingomyelinase, which converts sphingomyelin to ceramide and phosphorylcholine, also causes cholesterol accumulation in LE/Lys (Lloyd-Evans et al, 2008; Schloer et al, 2020a). Small compounds that inhibit sphingomyelinase are clinically approved, well-tolerated, and widely used to treat a broad spectrum of pathological conditions (Kornhuber et al, 2010). Along these lines, fluvoxamine was beneficial for COVID-19 patients in two recent studies (Kornhuber et al, 2022; Wen et al, 2022). A retrospective cohort study of almost 400,000 hospitalized COVID-19 patients suggested that prior use of antidepressant medications may reduce the likelihood of SARS-CoV-2 infection, hospitalization, and mortality. These associations between antidepressants and COVID-19 severity are most likely due to inhibition of acid sphingomyelinase (FIASMA) (Hoertel et al, 2022). Yet, fluvoxamine treatment of outpatients with mild to moderate COVID-19 did not improve the time to sustained recovery (Stewart et al, 2023), pointing at the need for further studies to evaluate the clinical effects of fluvoxamine in COVID-19.

Antidepressants including fluoxetine revealed reduced SARS-CoV-2 infection in Vero-E6 and Calu-3a cells and a decline in viral titers in murine lung tissue 3D ex vivo explants, which is supported by several retrospective and observational studies for fluoxetine and hydroxyzine over the course of COVID-19 (Schloer et al, 2021; Schloer et al, 2022). Strikingly, the combination of itraconazole or fluoxetine with remdesivir, a nucleotide analogue that inhibits the SARS-CoV-2 RNA polymerase, displayed stronger antiviral activities compared to monotherapy, indicating that targeting multiple regulatory pathways may be a valuable therapeutic strategy (Schloer et al, 2020a).

Other consequences associated with cholesterol accumulation in LE/Lys could potentiate capacity to reduce SARS-CoV-2 infection and propagation (Ballout et al, 2020). This includes the impaired proteolytic activity of cathepsin, which is critical for SARS-CoV-2 processing for cell entry in LE/Lys. The dysfunction of cathepsins appears related to cholesterol levels in this compartment, as clearance of accumulated cholesterol restored cathepsin B/L activity (Elrick & Lieberman, 2013; Ballout et al, 2020).

Blocked endolysosomal cholesterol efflux upon NPC1 inhibition causes cholesterol depletion in other cellular sites (Cubells et al, 2007; Musiol et al, 2013), such as the plasma membrane. Indeed, U18666A treatment or Rab7 inhibition, which also triggers LE/Lys-cholesterol accumulation (Meneses-Salas et al, 2020), reduced the number of released influenza virus progeny and lowered cholesterol in the viral envelope, both critical for the success of influenza infection (Musiol et al, 2013; Kuhnl et al, 2018). Yet, coronaviruses assemble at membranes of the ER Golgi intermediate compartment (ERGIC), followed by budding into the lumen and release via exocytosis of cargo vesicles (Stertz et al, 2007). As NPC1 inhibition also reduces cholesterol amounts in the ER, Golgi, and exocytic vesicles (Cubells et al, 2007; Reverter et al, 2014), one can speculate that blocked cholesterol export from LE/Lys may also lower cholesterol levels in ERGIC, with consequences for virus assembly and release.

Furthermore, with NPC1 inhibition lowering the amount of cholesterol-rich microdomains (Garver et al, 2002; Vainio et al, 2005; Cubells et al, 2007), one can envisage that lipid raft association of ACE2 could be compromised, with detrimental consequences for SARS-CoV-2 cell surface entry. In fact, type II pneumocytes, which are susceptible to SARS-CoV-2 infection and express ACE2 (Li et al, 2003; Hamming et al, 2004) depend on NPC1/2 to modulate the lipid composition of the pulmonary surfactant (Roszell et al, 2012; Rodriguez-Gil et al, 2019). Likewise, membrane proteases TMPRSS2 and ADAM17 are found predominantly in lipid rafts. As ADAM17 is elevated in NPC mutant cells, this might increase ACE2 shedding and counteract TMPRSS2, adding further protection against viral cell surface binding (Ballout et al, 2020).

Also, elevated levels of the two oxysterols 7-ketocholesterol and 25-hydroxycholesterol, both with potent antiviral activities (Massey [2006]; Wang et al [2020b]; Zang et al [2020]; Mao et al [2022]), accumulate in NPC1 deficiency (Porter et al, 2010).

Several other factors of the endocytic machinery in LE/Lys linked to cholesterol and NPC1 function could also emerge as druggable antiviral targets. This includes endosomal sorting complex required for transport complexes (Du et al, 2012, 2013), late endosomal annexins (Musiol et al, 2013; Kuhnl et al, 2018; Enrich et al, 2021), and Rab-GTPases (Zang et al, 2020) and their regulators and effectors (Meneses-Salas et al, 2020), but also proteins that link cholesterol and calcium balance in LE/Lys (Lloyd-Evans et al, 2008; Enrich et al, 2021).

Finally, it should be noted that despite these many antiviral properties mediated by LE/Lys-cholesterol accumulation, NPC deficiency also lowers cholesterol levels in the ER, which is associated with elevated SREBP2 activity (Kristiana et al, 2008; Meneses-Salas et al, 2020). The latter may activate the NOD-, LRR-, and pyrin domain–containing protein 3 (NLRP3) inflammasome and contribute to inflammatory reactions (see below). Future studies will have to address these potentially undesired outcomes.

### The prognostic value of systemic cholesterol levels in COVID-19

Infectious diseases are long known to be associated with low LDL and HDL levels (Deniz et al, 2007; Pirillo et al, 2015), probably reflecting a higher cellular demand for cholesterol to support viral propagation. Thus, LDL-, HDL-cholesterol, and total serum cholesterol levels were reduced in COVID-19 patients (Lee et al, 2020; Kim et al, 2021a; Usenko et al, 2023) and associated with a higher risk for infection, a more severe disease course, and death (Wang et al, 2021; Zhao et al, 2021). Indicating recovery after infection, LDL-, HDL-, and total cholesterol increased 3–6 mo after discharge from hospital (Li et al, 2021a). Notably, not all studies support an association of circulating cholesterol levels with SARS-CoV-2 infection (Caterino et al, 2021; Kukla et al, 2021; Ruscica et al, 2021; Lavis et al, 2022).

### Statin use and COVID-19 severity and outcome

**Figure 2.**
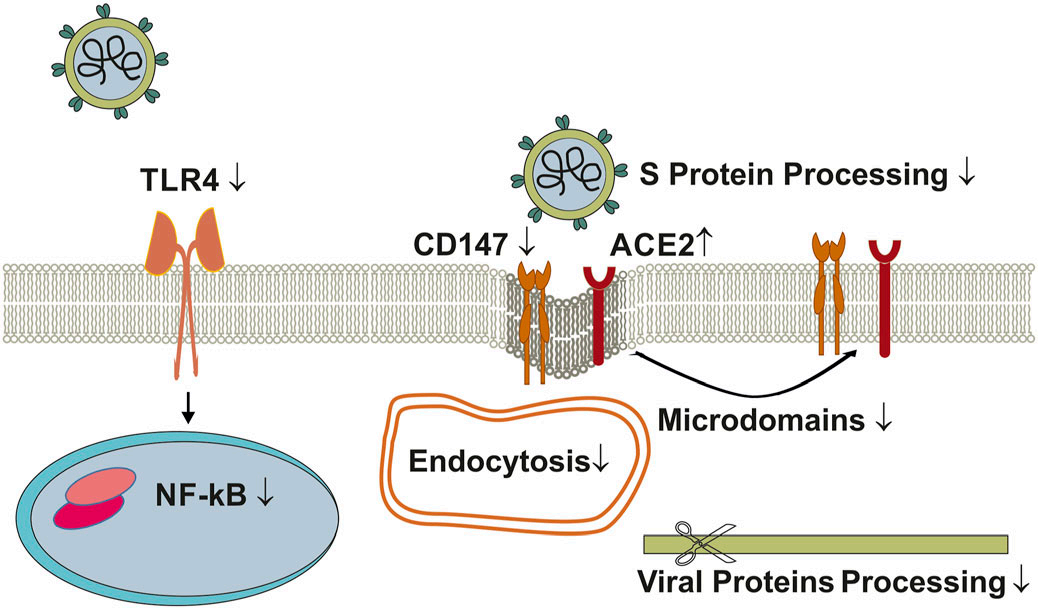
Effect of statins on SARS-CoV-2 infection. Statin-induced ACE2 displacement from cholesterol-rich lipid rafts and lowering of CD147 plasma membrane levels interfere with viral fusion and/or viral entry via endocytosis. Statins prevent ACE2 down-regulation upon SARS-CoV-2 infection and inhibit NF-kB and TLR4-regulated inflammatory pathways. Abbreviations: ACE2, angiotensin-converting enzyme 2; CD147, cluster of differentiation 147; NF-kB, nuclear factor kappa-light-chain-enhancer of activated B cells; S protein, spike protein; TLR4, Toll-like receptor 4.

Statins are widely prescribed drugs effectively lowering systemic LDL-cholesterol levels (Grewal & Buechler, 2022) that also substantially reduce cholesterol levels at the plasma membrane. The latter disturbs the formation of cholesterol-rich microdomains, and lipophilic statins (e.g., lovastatin, simvastatin, pitavastatin, atorvastatin) probably displace ACE2 from cholesterol-rich lipid rafts, which reduces its ability to serve as SARS-CoV-2 receptor (Bakillah et al, 2022; Fiore et al, 2022). Furthermore, statins can increase ACE2 protein levels (Fiore et al, 2022; Teixeira et al, 2022), which may antagonize the harmful effects of ACE2 down-regulation that occur after ACE2-mediated uptake of SARS-CoV-2 (Fig 2). Upon viral entry through this route, ACE2 suppression causes overactivation of the renin angiotensin system, associated with vasoconstriction, inflammation, edema and fibrosis, all of which contributing to COVID-19 disease severity (De Spiegeleer et al, 2020; Silhol et al, 2020).

In addition, in THP-1 macrophages, statins down-regulated the cell surface levels of the alternative SARS-CoV-2 receptor CD147, likely because of its intracellular retention (Sasidhar et al, 2017; Bakillah et al, 2022). Statins also down-regulate TMPRSS2, thereby reducing the processing of the viral S2 subunit after docking to ACE2, ultimately lowering fusion of the virus with the plasma membrane (Fiore et al, 2022). Furthermore, the anti-inflammatory activities of statins (Zivkovic et al, 2023) inhibiting NF-kB and TLR4-induced pathways (Behl et al, 2022) (Fig 2) lowered inflammation of SARS-CoV-2–infected cell lines such as Calu-3a cells, lung tissues, human monocytes, and neutrophils (Behl et al, 2022; Fiore et al, 2022; Teixeira et al, 2022; Zivkovic et al, 2023). The proposed S protein binding to human TLR4 (Choudhury & Mukherjee, 2020) might also be counteracted by the anti-inflammatory action of statins (Choudhury & Mukherjee, 2020) (Fig 2).

Although experimental evidence is still lacking, several recent in silico studies have proposed statins binding to proteins other than 3-hydroxy-3-methylglutaryl-CoA reductase, such as viral proteins and SARS-CoV-2 receptors, possibly affecting virus entry and propagation (Reiner et al, 2020; Ghosh et al, 2022) (Fig 2).

On the other hand, statin use before COVID-1 hospitalization was not reported to protect against a fatal outcome (Hariyanto & Kurniawan, 2020). The loss of geranyl-pyrophosphate precursors compromising protein prenylation are considered to explain the anti-inflammatory activities of statins (Smaldone et al, 2009). However, this also reduces production of coenzyme Q10, which is required for mitochondrial electron transport that drives the generation of ATP. Hence, statin-mediated reduction of coenzyme Q10 availability in patients with existing mitochondrial dysfunction may amplify the risk of poor COVID-19 outcomes (Golomb et al, 2023).

Hence, further research is still required to better define those patients in SARS-CoV-2–infected cohorts with mixed comorbidities that benefit most from statin use (Deshpande et al, 2015; Chen et al, 2018; Pienkos et al, 2023). The differential activity and pharmacokinetics of lipo- and hydrophilic statins also needs to be considered in this context.

### Other cholesterol-lowering drugs affecting COVID-19 disease

Besides statins, inhibitory antibodies against proprotein convertase subtilisin/kexin type 9 (PCSK9) are now used to treat hypercholesterinemia, increasing LDL receptor levels at the cell surface for enhanced LDL clearance (Sundararaman et al, 2021; Grewal & Buechler, 2022).

In addition, PCSK9 has several other functions, including its ability to promote ACE2 degradation (Essalmani et al, 2023), which might affect SARS-CoV-2 infectivity. On the other hand, in experimental models, PCSK9 inhibition reduced inflammation and improved survival in sepsis. SREBP2 as well as NF-kB, both transcription factors activated upon SARS-CoV-2 infection, can elevate PCSK9 expression (Grewal & Buechler, 2022; Elahi et al, 2023; Essalmani et al, 2023) and NF-kB or SREBP2 inhibitors normalized PCSK9 levels in PBMCs of COVID-19–infected patients (Lee et al, 2020).

Although not all studies support an association of PCSK9 expression and COVID-19 severity (Huang et al, 2021; Mester et al, 2023), the subcutaneous injection of evolocumab, a monoclonal inhibitory PCSK9 antibody, in patients with severe COVID-19 lowered the need for intubation and death. Although the lowering of circulating LDL-cholesterol levels may reduce cholesterol levels of peripheral cells and monocyte inflammation (Stiekema et al, 2021; Xie et al, 2022), the PCSK9 blockade improving COVID-19 outcomes cannot be easily explained by up-regulation of LDL receptor–mediated endocytosis and cellular cholesterol levels, a setting that would rather support COVID-19 infectivity. In fact, PCSK9 blockage reduced serum IL-6 levels, suggesting that lowering of circulating PCSK9 levels reduced inflammation, which may be partly due to increased ACE2 activity, which is associated with higher levels of the anti-inflammatory angiotensin 1–7 (Essalmani et al, 2023; Navarese et al, 2023). Along these lines, PCSK9-deficient mice did not display increased liver cholesterol and bile acid levels and may not cause cholesterol accumulation in different cells and tissues (Parker et al, 2013).

Ezetimibe blocks NPC1L1-mediated intestinal cholesterol absorption, thereby lowering systemic cholesterol levels. In addition, ezetimibe inhibits hepatic NPC1L1, up-regulates hepatic LDL receptor levels, and promotes biliary cholesterol excretion (Sudhop et al, 2009; Pramfalk et al, 2011; Jocher et al, 2022; Nejat et al, 2023). In several cell models, ezetimibe impaired viral entry (Chen et al, 2021), which correlates with reduced hospitalization of COVID-19 patients (Israel et al, 2021).

Pantethine, the dimer of pantetheine, an amid analogue of pantothenic acid (vitamin B5) inhibits fatty acid and cholesterol synthesis and lowered SARS-CoV-2 infection. This was accompanied by a greatly reduced expression of viral proteins and cellular release of viral particles. Antiviral activities of pantethine include down-regulation of host proteins needed for viral entry such as TMPRSS2 and inflammatory genes (Abou-Hamdan et al, 2023). Higher vitamin B5 intake correlated with a lower incidence of COVID-19 (Darand et al, 2022) and molecular docking studies suggested pantethine as a possible inhibitor of the main viral protease M^pro^ (Verma et al, 2022).

### SREBP2, multiple roles for the master regulator of sterol synthesis in COVID-19

The inappropriate innate immune response to SARS-CoV-2 is triggered by the activation of the NLRP3 inflammasome. As NLRP3 inhibition reduced COVID-19–like hyperinflammation and pathology in preclinical models, this has potential to treat severe SARS-CoV-2 complications (Diarimalala et al, 2023; Potere et al, 2023).

Upon statin-induced cholesterol depletion, NLRP3 activation and translocation to the Golgi apparatus occurs in an SREBP2-dependent manner, whereas cholesterol supplementation or SREBP2 blockage inhibited NLRP3 inflammasome activation and protected from inflammation (Guo et al, 2018).

Interestingly, despite elevated transcriptional activity of SREBP2 in COVID-19 disease (Swanson et al, 2019), a disconnect between SREBP2 activation in inflammation with cholesterol homeostasis was observed (Guo et al, 2018; Lee et al, 2020). Subsequent studies identified NF-kB inhibition to block SREBP2 activity in PBMCs of COVID-19 patients to reduce production of inflammatory cytokines. This inflammatory activity of SREBP2 was then validated in a rodent model, with pharmacological SREBP2 inhibition effectively lowering inflammation in sepsis (Lee et al, 2020).

**Figure 3.**
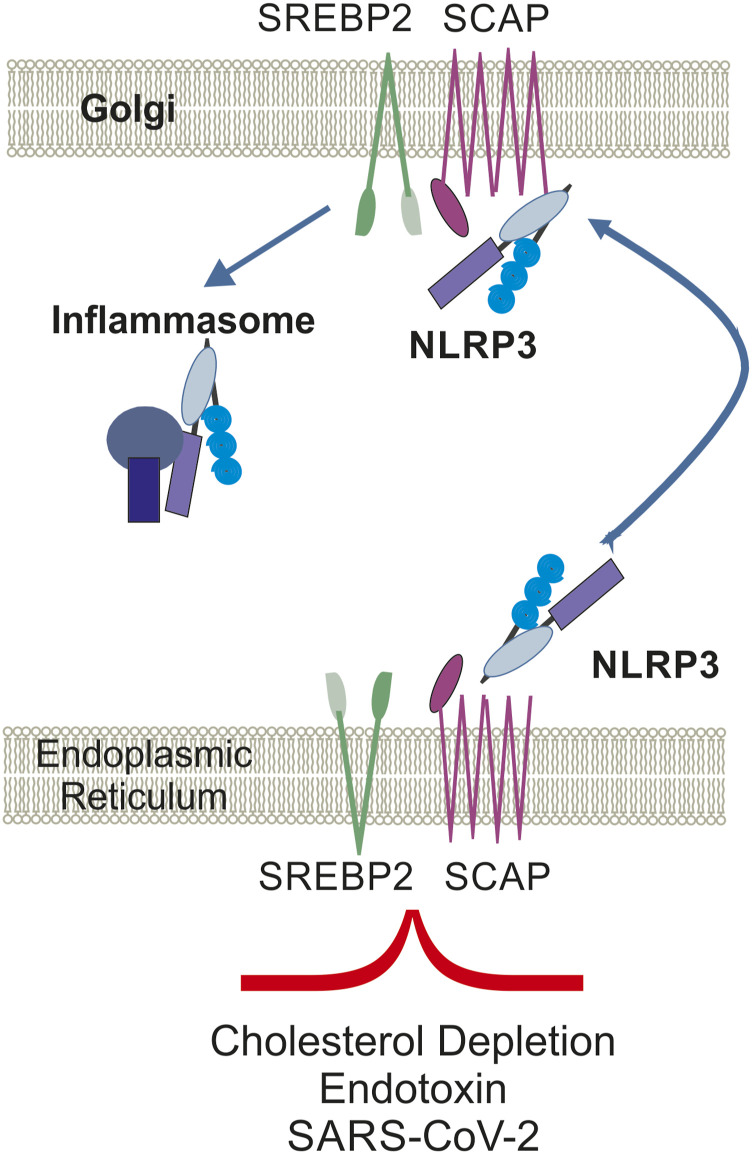
SREBP2 activation is related to inflammation in PBMCs of COVID-19 patients. SCAP-mediated escort of SREBP2 from the ER to the Golgi apparatus is required for the translocation of NLRP3 to the Golgi, enabling formation and activation of the inflammasome. Abbreviations: NF-kB, nuclear factor kappa-light-chain-enhancer of activated B cells; NLRP3, NOD-, LRR-, and pyrin domain–containing protein 3; PBMCs, peripheral blood mononuclear cells; SARS-CoV-2, severe acute respiratory syndrome coronavirus 2; SCAP, SREBP2 cleavage–activating protein; SREBP2, sterol regulatory element–binding protein 2.

Elevated TNF levels in COVID-19 patients (Han et al, 2020) can activate SREBP2, which induces interferon response and inflammatory gene expression in macrophages (Fig 3). Likewise, atorvastatin enhanced SREBP2-mediated up-regulation of inflammatory genes (Kusnadi et al, 2019). However, SARS-CoV-2–infected lung cancer cell lines A549 and Calu-3a revealed down-regulation of SREBP2 (Gomez Marti et al, 2021), indicating cell-dependent and differential outcome of SARS-CoV-2 infection on cholesterol homeostasis and SREBP2 activity.

## Conclusion

In this review, we described several key roles how cholesterol at the cell surface and in endolysosomes influences the efficacy of SARS-CoV-2 entry, providing opportunities for drugs targeting cholesterol-sensitive mechanism in these locations to reduce SARS-CoV-2 infectivity and COVID-19 disease severity. It would have gone beyond the scope of this review to cover the influence of cholesterol on many other aspects of the viral cycle, such as replication, assembly, and viral release, and we refer the reader to excellent review articles that cover cholesterol-sensitive mechanisms not only during SARS-CoV-2 surface recognition and cell entry but also viral replication, assembly, and release (Ballout et al, 2020; Fecchi et al, 2020; Tang et al, 2021b; Glitscher & Hildt, 2021; Palacios-Rapalo et al, 2021; Barrantes, 2022; Cesar-Silva et al, 2022; Kowalska et al, 2022; Wang et al, 2022, 2023; Ahmad et al, 2023).

Alike many other viruses, SARS-CoV-2 takes advantage of cellular cholesterol supply to gain entry and propagate in the host cell. Elevated cellular cholesterol levels support the generation of SARS-CoV-2 progeny for subsequent viral infections. These observations correlate with low serum cholesterol levels, disease severity, and death in COVID-19 patients. In many studies, patients using statins appear to experience less severe disease. Although statins may instigate SREBP2-dependent NLRP3 inflammasome activation in PBMCs, this does not appear to impact significantly in the various statin-related epidemiological studies in larger COVID-19 cohorts. The promising beneficial effects of inhibitory PCSK9 antibodies and other cholesterol-depleting drugs, which protected from viral infection in preclinical studies, have yet to be validated in patient cohorts. FDA-approved drugs that cause cholesterol accumulation in LE/Lys through NPC1 inhibition also provide opportunity to combat SARS-CoV-2 infectivity. With multiple cholesterol-sensitive events influencing SARS-CoV-2 infection, propagation, and release, further efforts to develop strategies that interfere with the availability of cholesterol for critical steps in the infectious cycle may be of benefit in the therapy of COVID-19. As mentioned earlier, age is a critical risk factor for COVID-19 severity. This correlates with cholesterol levels commonly increasing with age (Cutler et al, 2004; Wang et al, 2023) and disease (Xiong et al, 2008; Rudajev & Novotny, 2022). On the other hand, children have comparatively lower cholesterol levels (Cutler et al, 2004) and a better outcome than the elderly when infected with SARS-CoV-2 (Kang et al, 2022). This age-related correlation between cholesterol levels and disease severity supports strategies aiming to lower cholesterol in the elderly to reduce COVID-19 severity.

Cholesterol plays a crucial role during multiple steps in the life cycle of almost all viruses. Thus, manipulating cholesterol homeostasis could potentially serve as a broad-spectrum antiviral strategy. As outlined in this review, rather than reducing cholesterol levels globally, the key to cholesterol-related antiviral strategies may require disruption of cholesterol handling in specific cellular organelles where it is needed for viral entry, replication, and propagation (Glitscher & Hildt, 2021). Endolysosomal cholesterol homeostasis has been recognized as a potential target for antiviral treatments against a variety of viruses, including SARS-CoV-2, but also influenza A and Ebola viruses. In the case of Ebola, NPC1 is hijacked by the virus to cross the endolysosomal membrane for cell entry. Hence, drugs interfering with NPC1 activity and endolysosomal cholesterol levels such as itraconazole and fluoxetine have potential to deliver broad antiviral effects (Glitscher & Hildt, 2021; Kummer et al, 2022). Although the availability of these tools offer promising treatment options against viral infections, further research is still needed to fully understand the impact of interfering with cholesterol transport in cells and to assess adverse effects of these drugs (Glitscher & Hildt, 2021).

### Author Contributions

T Grewal: writing—review and editing.
MKL Nguyen: writing—review and editing.
C Buechler: writing—original draft and writing—review and editing.

## Supplementary Material

Reviewer comments

## References

[bib1] Abou-Hamdan M, Saleh R, Mani S, Dournaud P, Metifiot M, Blondot ML, Andreola ML, Abdel-Sater F, De Reggi M, Gressens P, (2023) Potential antiviral effects of pantethine against SARS-CoV-2. Sci Rep 13: 2237. 10.1038/s41598-023-29245-0 36754974 PMC9906591

[bib2] Ahmad I, Fatemi SN, Ghaheri M, Rezvani A, Khezri DA, Natami M, Yasamineh S, Gholizadeh O, Bahmanyar Z (2023) An overview of the role of Niemann-pick C1 (NPC1) in viral infections and inhibition of viral infections through NPC1 inhibitor. Cell Commun Signal 21: 352. 10.1186/s12964-023-01376-x 38098077 PMC10722723

[bib3] Al-Horani RA, Kar S, Aliter KF (2020) Potential anti-COVID-19 therapeutics that block the early stage of the viral life cycle: Structures, mechanisms, and clinical trials. Int J Mol Sci 21: 5224. 10.3390/ijms21155224 32718020 PMC7432953

[bib4] Alkafaas SS, Abdallah AM, Ghosh S, Loutfy SA, Elkafas SS, Abdel Fattah NF, Hessien M (2023) Insight into the role of clathrin-mediated endocytosis inhibitors in SARS-CoV-2 infection. Rev Med Virol 33: e2403. 10.1002/rmv.2403 36345157 PMC9877911

[bib5] Axfors C, Schmitt AM, Janiaud P, Van’t Hooft J, Abd-Elsalam S, Abdo EF, Abella BS, Akram J, Amaravadi RK, Angus DC, (2021) Mortality outcomes with hydroxychloroquine and chloroquine in COVID-19 from an international collaborative meta-analysis of randomized trials. Nat Commun 12: 2349. 10.1038/s41467-021-22446-z 33859192 PMC8050319

[bib6] Babayigit C, Kokturk N, Kul S, Cetinkaya PD, Atis Nayci S, Argun Baris S, Karcioglu O, Aysert P, Irmak I, Akbas Yuksel A, (2022) The association of antiviral drugs with COVID-19 morbidity: The retrospective analysis of a nationwide COVID-19 cohort. Front Med (Lausanne) 9: 894126. 10.3389/fmed.2022.894126 36117966 PMC9471091

[bib7] Bakillah A, Hejji FA, Almasaud A, Jami HA, Hawwari A, Qarni AA, Iqbal J, Alharbi NK (2022) Lipid raft integrity and cellular cholesterol homeostasis are critical for SARS-CoV-2 entry into cells. Nutrients 14: 3417. 10.3390/nu14163417 36014919 PMC9415163

[bib8] Ballout RA, Sviridov D, Bukrinsky MI, Remaley AT (2020) The lysosome: A potential juncture between SARS-CoV-2 infectivity and Niemann-Pick disease type C, with therapeutic implications. FASEB J 34: 7253–7264. 10.1096/fj.202000654R 32367579 PMC7383733

[bib9] Barrantes FJ (2022) The constellation of cholesterol-dependent processes associated with SARS-CoV-2 infection. Prog Lipid Res 87: 101166. 10.1016/j.plipres.2022.101166 35513161 PMC9059347

[bib10] Bayati A, Kumar R, Francis V, McPherson PS (2021) SARS-CoV-2 infects cells after viral entry via clathrin-mediated endocytosis. J Biol Chem 296: 100306. 10.1016/j.jbc.2021.100306 33476648 PMC7816624

[bib11] Bean J, Kuri-Cervantes L, Pennella M, Betts MR, Meyer NJ, Hassan WM (2023) Multivariate indicators of disease severity in COVID-19. Sci Rep 13: 5145. 10.1038/s41598-023-31683-9 36991002 PMC10054197

[bib12] Behl T, Kaur I, Aleya L, Sehgal A, Singh S, Sharma N, Bhatia S, Al-Harrasi A, Bungau S (2022) CD147-spike protein interaction in COVID-19: Get the ball rolling with a novel receptor and therapeutic target. Sci Total Environ 808: 152072. 10.1016/j.scitotenv.2021.152072 34863742 PMC8634688

[bib13] Bestle D, Heindl MR, Limburg H, Van Lam van T, Pilgram O, Moulton H, Stein DA, Hardes K, Eickmann M, Dolnik O, (2020) TMPRSS2 and furin are both essential for proteolytic activation of SARS-CoV-2 in human airway cells. Life Sci Alliance 3: e202000786. 10.26508/lsa.202000786 32703818 PMC7383062

[bib14] Bezerra BB, Silva G, Coelho SVA, Correa IA, Souza MRM, Macedo KVG, Matos BM, Tanuri A, Matassoli FL, Costa LJD, (2022) Hydroxypropyl-beta-cyclodextrin (HP-BCD) inhibits SARS-CoV-2 replication and virus-induced inflammatory cytokines. Antivir Res 205: 105373. 10.1016/j.antiviral.2022.105373 35798224 PMC9250893

[bib15] Blair HA (2023) Nirmatrelvir plus ritonavir in COVID-19: A profile of its use. Drugs Ther Perspect 39: 41–47. 10.1007/s40267-022-00971-1 36532315 PMC9734504

[bib16] Booth A, Reed AB, Ponzo S, Yassaee A, Aral M, Plans D, Labrique A, Mohan D (2021) Population risk factors for severe disease and mortality in COVID-19: A global systematic review and meta-analysis. PLoS One 16: e0247461. 10.1371/journal.pone.0247461 33661992 PMC7932512

[bib17] Carette JE, Raaben M, Wong AC, Herbert AS, Obernosterer G, Mulherkar N, Kuehne AI, Kranzusch PJ, Griffin AM, Ruthel G, (2011) Ebola virus entry requires the cholesterol transporter Niemann-Pick C1. Nature 477: 340–343. 10.1038/nature10348 21866103 PMC3175325

[bib18] Caterino M, Gelzo M, Sol S, Fedele R, Annunziata A, Calabrese C, Fiorentino G, D’Abbraccio M, Dell’Isola C, Fusco FM, (2021) Dysregulation of lipid metabolism and pathological inflammation in patients with COVID-19. Sci Rep 11: 2941. 10.1038/s41598-021-82426-7 33536486 PMC7859398

[bib19] Cenedella RJ, Jacob R, Borchman D, Tang D, Neely AR, Samadi A, Mason RP, Sexton P (2004) Direct perturbation of lens membrane structure may contribute to cataracts caused by U18666A, an oxidosqualene cyclase inhibitor. J Lipid Res 45: 1232–1241. 10.1194/jlr.M300469-JLR200 15102886

[bib20] Cesar-Silva D, Pereira-Dutra FS, Moraes Giannini AL, Jacques G de Almeida C (2022) The endolysosomal system: The acid test for SARS-CoV-2. Int J Mol Sci 23: 4576. 10.3390/ijms23094576 35562967 PMC9105036

[bib21] Chen M, Ji M, Si X (2018) The effects of statin therapy on mortality in patients with sepsis: A meta-analysis of randomized trials. Medicine (Baltimore) 97: e11578. 10.1097/MD.0000000000011578 30075526 PMC6081129

[bib22] Chen F, Shi Q, Pei F, Vogt A, Porritt RA, Garcia G, Jr, Gomez AC, Cheng MH, Schurdak ME, Liu B, (2021) A systems-level study reveals host-targeted repurposable drugs against SARS-CoV-2 infection. Mol Syst Biol 17: e10239. 10.15252/msb.202110239 34339582 PMC8328275

[bib23] Cheng S, Zhao Y, Wang F, Chen Y, Kaminga AC, Xu H (2021) Comorbidities' potential impacts on severe and non-severe patients with COVID-19: A systematic review and meta-analysis. Medicine (Baltimore) 100: e24971. 10.1097/MD.0000000000024971 33761654 PMC9281964

[bib24] Choudhury A, Mukherjee S (2020) In silico studies on the comparative characterization of the interactions of SARS-CoV-2 spike glycoprotein with ACE-2 receptor homologs and human TLRs. J Med Virol 92: 2105–2113. 10.1002/jmv.25987 32383269 PMC7267663

[bib25] Cote M, Misasi J, Ren T, Bruchez A, Lee K, Filone CM, Hensley L, Li Q, Ory D, Chandran K, (2011) Small molecule inhibitors reveal Niemann-Pick C1 is essential for Ebola virus infection. Nature 477: 344–348. 10.1038/nature10380 21866101 PMC3230319

[bib26] Cubells L, Vila de Muga S, Tebar F, Wood P, Evans R, Ingelmo-Torres M, Calvo M, Gaus K, Pol A, Grewal T, (2007) Annexin A6-induced alterations in cholesterol transport and caveolin export from the Golgi complex. Traffic 8: 1568–1589. 10.1111/j.1600-0854.2007.00640.x 17822395 PMC3003291

[bib27] Cutler RG, Kelly J, Storie K, Pedersen WA, Tammara A, Hatanpaa K, Troncoso JC, Mattson MP (2004) Involvement of oxidative stress-induced abnormalities in ceramide and cholesterol metabolism in brain aging and Alzheimer's disease. Proc Natl Acad Sci U S A 101: 2070–2075. 10.1073/pnas.0305799101 14970312 PMC357053

[bib28] Darand M, Hassanizadeh S, Martami F, Shams S, Mirzaei M, Hosseinzadeh M (2022) The association between B vitamins and the risk of COVID-19. Br J Nutr 130: 155–163. 10.1017/s0007114522003075 36348570

[bib29] De Spiegeleer A, Bronselaer A, Teo JT, Byttebier G, De Tre G, Belmans L, Dobson R, Wynendaele E, Van De Wiele C, Vandaele F, (2020) The effects of ARBs, ACEis, and statins on clinical outcomes of COVID-19 infection among nursing home residents. J Am Med Dir Assoc 21: 909–914.e2. 10.1016/j.jamda.2020.06.018 32674818 PMC7294267

[bib30] Deniz O, Gumus S, Yaman H, Ciftci F, Ors F, Cakir E, Tozkoparan E, Bilgic H, Ekiz K (2007) Serum total cholesterol, HDL-C and LDL-C concentrations significantly correlate with the radiological extent of disease and the degree of smear positivity in patients with pulmonary tuberculosis. Clin Biochem 40: 162–166. 10.1016/j.clinbiochem.2006.10.015 17217941

[bib31] Deshpande A, Pasupuleti V, Rothberg MB (2015) Statin therapy and mortality from sepsis: A meta-analysis of randomized trials. Am J Med 128: 410–417.e1. 10.1016/j.amjmed.2014.10.057 25526798

[bib32] Diarimalala RO, Wei Y, Hu D, Hu K (2023) Inflammasomes during SARS-CoV-2 infection and development of their corresponding inhibitors. Front Cell Infect Microbiol 13: 1218039. 10.3389/fcimb.2023.1218039 37360532 PMC10288989

[bib33] Du X, Kazim AS, Brown AJ, Yang H (2012) An essential role of Hrs/Vps27 in endosomal cholesterol trafficking. Cell Rep 1: 29–35. 10.1016/j.celrep.2011.10.004 22832105

[bib34] Du X, Kazim AS, Dawes IW, Brown AJ, Yang H (2013) The AAA ATPase VPS4/SKD1 regulates endosomal cholesterol trafficking independently of ESCRT-III. Traffic 14: 107–119. 10.1111/tra.12015 23009658

[bib35] Elahi R, Hozhabri S, Moradi A, Siahmansouri A, Jahani Maleki A, Esmaeilzadeh A (2023) Targeting the cGAS-STING pathway as an inflammatory crossroad in coronavirus disease 2019 (COVID-19). Immunopharmacol Immunotoxicol 45: 639–649. 10.1080/08923973.2023.2215405 37335770

[bib36] Elrick MJ, Lieberman AP (2013) Autophagic dysfunction in a lysosomal storage disorder due to impaired proteolysis. Autophagy 9: 234–235. 10.4161/auto.22501 23086309 PMC3552886

[bib37] Enrich C, Rentero C, Hierro A, Grewal T (2015) Role of cholesterol in SNARE-mediated trafficking on intracellular membranes. J Cell Sci 128: 1071–1081. 10.1242/jcs.164459 25653390

[bib38] Enrich C, Lu A, Tebar F, Rentero C, Grewal T (2021) Annexins bridging the gap: Novel roles in membrane contact site formation. Front Cell Dev Biol 9: 797949. 10.3389/fcell.2021.797949 35071237 PMC8770259

[bib39] Essalmani R, Andreo U, Evagelidis A, Le Devehat M, Pereira Ramos OH, Fruchart Gaillard C, Susan-Resiga D, Cohen EA, Seidah NG (2023) SKI-1/S1P facilitates SARS-CoV-2 spike induced cell-to-cell fusion via activation of SREBP-2 and metalloproteases, whereas PCSK9 enhances the degradation of ACE2. Viruses 15: 360. 10.3390/v15020360 36851576 PMC9959508

[bib40] Fecchi K, Anticoli S, Peruzzu D, Iessi E, Gagliardi MC, Matarrese P, Ruggieri A (2020) Coronavirus interplay with lipid rafts and autophagy unveils promising therapeutic targets. Front Microbiol 11: 1821. 10.3389/fmicb.2020.01821 32849425 PMC7431668

[bib41] Fiore D, Proto MC, Franceschelli S, Pascale M, Bifulco M, Gazzerro P (2022) In vitro evidence of statins' protective role against COVID-19 hallmarks. Biomedicines 10: 2123. 10.3390/biomedicines10092123 36140223 PMC9495908

[bib42] Garver WS, Krishnan K, Gallagos JR, Michikawa M, Francis GA, Heidenreich RA (2002) Niemann-Pick C1 protein regulates cholesterol transport to the trans-Golgi network and plasma membrane caveolae. J Lipid Res 43: 579–589. 10.1016/s0022-2275(20)31487-5 11907140

[bib43] Ghosh D, Ghosh Dastidar D, Roy K, Ghosh A, Mukhopadhyay D, Sikdar N, Biswas NK, Chakrabarti G, Das A (2022) Computational prediction of the molecular mechanism of statin group of drugs against SARS-CoV-2 pathogenesis. Sci Rep 12: 6241. 10.1038/s41598-022-09845-y 35422113 PMC9009757

[bib44] Gkouskou K, Vasilogiannakopoulou T, Andreakos E, Davanos N, Gazouli M, Sanoudou D, Eliopoulos AG (2021) COVID-19 enters the expanding network of apolipoprotein E4-related pathologies. Redox Biol 41: 101938. 10.1016/j.redox.2021.101938 33730676 PMC7943392

[bib45] Glitscher M, Hildt E (2021) Endosomal cholesterol in viral infections - a common denominator? Front Physiol 12: 750544. 10.3389/fphys.2021.750544 34858206 PMC8632007

[bib46] Golomb BA, Han JH, Langsjoen PH, Dinkeloo E, Zemljic-Harpf AE (2023) Statin use in relation to COVID-19 and other respiratory infections: Muscle and other considerations. J Clin Med 12: 4659. 10.3390/jcm12144659 37510774 PMC10380486

[bib47] Gomez Marti JL, Wells A, Brufsky AM (2021) Dysregulation of the mevalonate pathway during SARS-CoV-2 infection: An in silico study. J Med Virol 93: 2396–2405. 10.1002/jmv.26743 33331649 PMC9553089

[bib48] Gong Y, Qin S, Dai L, Tian Z (2021) The glycosylation in SARS-CoV-2 and its receptor ACE2. Signal Transduct Target Ther 6: 396. 10.1038/s41392-021-00809-8 34782609 PMC8591162

[bib49] Grewal T, Buechler C (2022) Emerging insights on the diverse roles of proprotein convertase subtilisin/kexin type 9 (PCSK9) in chronic liver diseases: Cholesterol metabolism and beyond. Int J Mol Sci 23: 1070. 10.3390/ijms23031070 35162992 PMC8834914

[bib50] Guo C, Chi Z, Jiang D, Xu T, Yu W, Wang Z, Chen S, Zhang L, Liu Q, Guo X, (2018) Cholesterol homeostatic regulator SCAP-SREBP2 integrates NLRP3 inflammasome activation and cholesterol biosynthetic signaling in macrophages. Immunity 49: 842–856.e7. 10.1016/j.immuni.2018.08.021 30366764

[bib51] Hamming I, Timens W, Bulthuis ML, Lely AT, Navis G, van Goor H (2004) Tissue distribution of ACE2 protein, the functional receptor for SARS coronavirus. A first step in understanding SARS pathogenesis. J Pathol 203: 631–637. 10.1002/path.1570 15141377 PMC7167720

[bib52] Han H, Ma Q, Li C, Liu R, Zhao L, Wang W, Zhang P, Liu X, Gao G, Liu F, (2020) Profiling serum cytokines in COVID-19 patients reveals IL-6 and IL-10 are disease severity predictors. Emerg Microbes Infect 9: 1123–1130. 10.1080/22221751.2020.1770129 32475230 PMC7473317

[bib53] Hariyanto TI, Kurniawan A (2020) Statin therapy did not improve the in-hospital outcome of coronavirus disease 2019 (COVID-19) infection. Diabetes Metab Syndr 14: 1613–1615. 10.1016/j.dsx.2020.08.023 32882643 PMC7448951

[bib54] Hikmet F, Mear L, Edvinsson A, Micke P, Uhlen M, Lindskog C (2020) The protein expression profile of ACE2 in human tissues. Mol Syst Biol 16: e9610. 10.15252/msb.20209610 32715618 PMC7383091

[bib55] Hoertel N, Sanchez-Rico M, Kornhuber J, Gulbins E, Reiersen AM, Lenze EJ, Fritz BA, Jalali F, Mills EJ, Cougoule C, (2022) Antidepressant use and its association with 28-day mortality in inpatients with SARS-CoV-2: Support for the FIASMA model against COVID-19. J Clin Med 11: 5882. 10.3390/jcm11195882 36233753 PMC9572995

[bib56] Hoffmann M, Kleine-Weber H, Schroeder S, Kruger N, Herrler T, Erichsen S, Schiergens TS, Herrler G, Wu NH, Nitsche A, (2020) SARS-CoV-2 cell entry depends on ACE2 and TMPRSS2 and is blocked by a clinically proven protease inhibitor. Cell 181: 271–280.e8. 10.1016/j.cell.2020.02.052 32142651 PMC7102627

[bib57] Huang IC, Bosch BJ, Li F, Li W, Lee KH, Ghiran S, Vasilieva N, Dermody TS, Harrison SC, Dormitzer PR, (2006) SARS coronavirus, but not human coronavirus NL63, utilizes cathepsin L to infect ACE2-expressing cells. J Biol Chem 281: 3198–3203. 10.1074/jbc.M508381200 16339146 PMC8010168

[bib58] Huang W, Xiao J, Ji J, Chen L (2021) Association of lipid-lowering drugs with COVID-19 outcomes from a Mendelian randomization study. Elife 10: e73873. 10.7554/eLife.73873 34866576 PMC8709572

[bib59] Israel A, Schaffer AA, Cicurel A, Cheng K, Sinha S, Schiff E, Feldhamer I, Tal A, Lavie G, Ruppin E (2021) Identification of drugs associated with reduced severity of COVID-19 - a case-control study in a large population. Elife 10: e68165. 10.7554/eLife.68165 34313216 PMC8321549

[bib60] Jocher G, Grass V, Tschirner SK, Riepler L, Breimann S, Kaya T, Oelsner M, Hamad MS, Hofmann LI, Blobel CP, (2022) ADAM10 and ADAM17 promote SARS-CoV-2 cell entry and spike protein-mediated lung cell fusion. EMBO Rep 23: e54305. 10.15252/embr.202154305 35527514 PMC9171409

[bib61] Kang YL, Chou YY, Rothlauf PW, Liu Z, Soh TK, Cureton D, Case JB, Chen RE, Diamond MS, Whelan SPJ, (2020) Inhibition of PIKfyve kinase prevents infection by Zaire ebolavirus and SARS-CoV-2. Proc Natl Acad Sci U S A 117: 20803–20813. 10.1073/pnas.2007837117 32764148 PMC7456157

[bib62] Kang CK, Shin HM, Park WB, Kim HR (2022) Why are children less affected than adults by severe acute respiratory syndrome coronavirus 2 infection? Cell Mol Immunol 19: 555–557. 10.1038/s41423-022-00857-2 35332299 PMC8943348

[bib63] Kim SW, Kang HJ, Jhon M, Kim JW, Lee JY, Walker AJ, Agustini B, Kim JM, Berk M (2019) Statins and inflammation: New therapeutic opportunities in psychiatry. Front Psychiatry 10: 103. 10.3389/fpsyt.2019.00103 30890971 PMC6413672

[bib64] Kim H, Lee HS, Ahn JH, Hong KS, Jang JG, An J, Mun YH, Yoo SY, Choi YJ, Yun MY, (2021a) Lung-selective 25-hydroxycholesterol nanotherapeutics as a suppressor of COVID-19-associated cytokine storm. Nano Today 38: 101149. 10.1016/j.nantod.2021.101149 33846686 PMC8026257

[bib65] Kim TY, Jeon S, Jang Y, Gotina L, Won J, Ju YH, Kim S, Jang MW, Won W, Park MG, (2021b) Platycodin D, a natural component of Platycodon grandiflorum, prevents both lysosome- and TMPRSS2-driven SARS-CoV-2 infection by hindering membrane fusion. Exp Mol Med 53: 956–972. 10.1038/s12276-021-00624-9 34035463 PMC8143993

[bib66] Kornhuber J, Tripal P, Reichel M, Muhle C, Rhein C, Muehlbacher M, Groemer TW, Gulbins E (2010) Functional inhibitors of acid sphingomyelinase (FIASMAs): A novel pharmacological group of drugs with broad clinical applications. Cell Physiol Biochem 26: 9–20. 10.1159/000315101 20502000

[bib67] Kornhuber J, Hoertel N, Gulbins E (2022) The acid sphingomyelinase/ceramide system in COVID-19. Mol Psychiatry 27: 307–314. 10.1038/s41380-021-01309-5 34608263 PMC8488928

[bib68] Kovacs T, Kurtan K, Varga Z, Nagy P, Panyi G, Zakany F (2023) Veklury® (remdesivir) formulations inhibit initial membrane-coupled events of SARS-CoV-2 infection due to their sulfobutylether-β-cyclodextrin content. Br J Pharmacol 180: 2064–2084. 10.1111/bph.16063 36848880

[bib69] Kowalska K, Sabatowska Z, Forycka J, Mlynarska E, Franczyk B, Rysz J (2022) The influence of SARS-CoV-2 infection on lipid metabolism-the potential use of lipid-lowering agents in COVID-19 management. Biomedicines 10: 2320. 10.3390/biomedicines10092320 36140421 PMC9496398

[bib70] Kreutzberger AJB, Sanyal A, Saminathan A, Bloyet LM, Stumpf S, Liu Z, Ojha R, Patjas MT, Geneid A, Scanavachi G, (2022) SARS-CoV-2 requires acidic pH to infect cells. Proc Natl Acad Sci U S A 119: e2209514119. 10.1073/pnas.2209514119 36048924 PMC9499588

[bib71] Kristiana I, Yang H, Brown AJ (2008) Different kinetics of cholesterol delivery to components of the cholesterol homeostatic machinery: Implications for cholesterol trafficking to the endoplasmic reticulum. Biochim Biophys Acta 1781: 724–730. 10.1016/j.bbalip.2008.08.006 18838129

[bib72] Kuhnl A, Musiol A, Heitzig N, Johnson DE, Ehrhardt C, Grewal T, Gerke V, Ludwig S, Rescher U (2018) Late endosomal/lysosomal cholesterol accumulation is a host cell-protective mechanism inhibiting endosomal escape of influenza A virus. mBio 9: e01345-18. 10.1128/mBio.01345-18 30042202 PMC6058292

[bib73] Kukla M, Menzyk T, Dembinski M, Winiarski M, Garlicki A, Bociaga-Jasik M, Skonieczna M, Hudy D, Maziarz B, Kusnierz-Cabala B, (2021) Anti-inflammatory adipokines: Chemerin, vaspin, omentin concentrations and SARS-CoV-2 outcomes. Sci Rep 11: 21514. 10.1038/s41598-021-00928-w 34728695 PMC8563971

[bib74] Kumar R, Sharma A, Srivastava JK, Siddiqui MH, Uddin MS, Aleya L (2021) Hydroxychloroquine in COVID-19: Therapeutic promises, current status, and environmental implications. Environ Sci Pollut Res Int 28: 40431–40444. 10.1007/s11356-020-12200-1 33447984 PMC7808930

[bib75] Kummer S, Lander A, Goretzko J, Kirchoff N, Rescher U, Schloer S (2022) Pharmacologically induced endolysosomal cholesterol imbalance through clinically licensed drugs itraconazole and fluoxetine impairs Ebola virus infection in vitro. Emerg Microbes Infect 11: 195–207. 10.1080/22221751.2021.2020598 34919035 PMC8745396

[bib76] Kuo CL, Pilling LC, Atkins JL, Masoli JAH, Delgado J, Kuchel GA, Melzer D (2020) APOE e4 Genotype Predicts Severe COVID-19 in the UK Biobank Community Cohort. J Gerontol A Biol Sci Med Sci 75: 2231–2232. 10.1093/gerona/glaa131 32451547 PMC7314139

[bib77] Kusnadi A, Park SH, Yuan R, Pannellini T, Giannopoulou E, Oliver D, Lu T, Park-Min KH, Ivashkiv LB (2019) The cytokine TNF promotes transcription factor SREBP activity and binding to inflammatory genes to activate macrophages and limit tissue repair. Immunity 51: 241–257.e9. 10.1016/j.immuni.2019.06.005 31303399 PMC6709581

[bib78] Lachmann RH, te Vruchte D, Lloyd-Evans E, Reinkensmeier G, Sillence DJ, Fernandez-Guillen L, Dwek RA, Butters TD, Cox TM, Platt FM (2004) Treatment with miglustat reverses the lipid-trafficking defect in Niemann-Pick disease type C. Neurobiol Dis 16: 654–658. 10.1016/j.nbd.2004.05.002 15262277

[bib79] Lavis P, Morra S, Orte Cano C, Albayrak N, Corbiere V, Olislagers V, Dauby N, Del Marmol V, Marchant A, Decaestecker C, (2022) Chemerin plasma levels are increased in COVID-19 patients and are an independent risk factor of mortality. Front Immunol 13: 941663. 10.3389/fimmu.2022.941663 36032171 PMC9412239

[bib80] Lee W, Ahn JH, Park HH, Kim HN, Kim H, Yoo Y, Shin H, Hong KS, Jang JG, Park CG, (2020) COVID-19-activated SREBP2 disturbs cholesterol biosynthesis and leads to cytokine storm. Signal Transduct Target Ther 5: 186. 10.1038/s41392-020-00292-7 32883951 PMC7471497

[bib81] Li W, Moore MJ, Vasilieva N, Sui J, Wong SK, Berne MA, Somasundaran M, Sullivan JL, Luzuriaga K, Greenough TC, (2003) Angiotensin-converting enzyme 2 is a functional receptor for the SARS coronavirus. Nature 426: 450–454. 10.1038/nature02145 14647384 PMC7095016

[bib82] Li GM, Li YG, Yamate M, Li SM, Ikuta K (2007) Lipid rafts play an important role in the early stage of severe acute respiratory syndrome-coronavirus life cycle. Microbes Infect 9: 96–102. 10.1016/j.micinf.2006.10.015 17194611 PMC7110773

[bib83] Li G, Du L, Cao X, Wei X, Jiang Y, Lin Y, Nguyen V, Tan W, Wang H (2021a) Follow-up study on serum cholesterol profiles and potential sequelae in recovered COVID-19 patients. BMC Infect Dis 21: 299. 10.1186/s12879-021-05984-1 33761881 PMC7989719

[bib84] Li X, Zhu W, Fan M, Zhang J, Peng Y, Huang F, Wang N, He L, Zhang L, Holmdahl R, (2021b) Dependence of SARS-CoV-2 infection on cholesterol-rich lipid raft and endosomal acidification. Comput Struct Biotechnol J 19: 1933–1943. 10.1016/j.csbj.2021.04.001 33850607 PMC8028701

[bib85] Liesenborghs L, Spriet I, Jochmans D, Belmans A, Gyselinck I, Teuwen LA, Ter Horst S, Dreesen E, Geukens T, Engelen MM, (2021) Itraconazole for COVID-19: Preclinical studies and a proof-of-concept randomized clinical trial. EBioMedicine 66: 103288. 10.1016/j.ebiom.2021.103288 33752127 PMC7979145

[bib86] Liu Y, Pan Y, Yin Y, Chen W, Li X (2021) Association of dyslipidemia with the severity and mortality of coronavirus disease 2019 (COVID-19): A meta-analysis. Virol J 18: 157. 10.1186/s12985-021-01604-1 34315474 PMC8314261

[bib87] Lloyd-Evans E, Morgan AJ, He X, Smith DA, Elliot-Smith E, Sillence DJ, Churchill GC, Schuchman EH, Galione A, Platt FM (2008) Niemann-Pick disease type C1 is a sphingosine storage disease that causes deregulation of lysosomal calcium. Nat Med 14: 1247–1255. 10.1038/nm.1876 18953351

[bib88] Lu Y, Liu DX, Tam JP (2008) Lipid rafts are involved in SARS-CoV entry into Vero E6 cells. Biochem Biophys Res Commun 369: 344–349. 10.1016/j.bbrc.2008.02.023 18279660 PMC7092920

[bib89] Makhoul E, Aklinski JL, Miller J, Leonard C, Backer S, Kahar P, Parmar MS, Khanna D (2022) A review of COVID-19 in relation to metabolic syndrome: Obesity, hypertension, diabetes, and dyslipidemia. Cureus 14: e27438. 10.7759/cureus.27438 36051728 PMC9420458

[bib90] Mansouri V, Tavasoli AR, Khodarahmi M, Dakkali MS, Daneshfar S, Ashrafi MR, Heidari M, Hosseinpour S, Sharifianjazi F, Bemanalizadeh M (2023) Efficacy and safety of miglustat in the treatment of GM2 gangliosidosis: A systematic review. Eur J Neurol 30: 2919–2945. 10.1111/ene.15871 37209042

[bib91] Mao S, Ren J, Xu Y, Lin J, Pan C, Meng Y, Xu N (2022) Studies in the antiviral molecular mechanisms of 25-hydroxycholesterol: Disturbing cholesterol homeostasis and post-translational modification of proteins. Eur J Pharmacol 926: 175033. 10.1016/j.ejphar.2022.175033 35598845 PMC9119167

[bib92] Massey JB (2006) Membrane and protein interactions of oxysterols. Curr Opin Lipidol 17: 296–301. 10.1097/01.mol.0000226123.17629.ab 16680036

[bib93] Meher G, Bhattacharjya S, Chakraborty H (2023) Membrane cholesterol regulates the oligomerization and fusogenicity of SARS-CoV fusion peptide: Implications in viral entry. Phys Chem Chem Phys 25: 7815–7824. 10.1039/d2cp04741a 36857640

[bib94] Meneses-Salas E, Garcia-Melero A, Kanerva K, Blanco-Munoz P, Morales-Paytuvi F, Bonjoch J, Casas J, Egert A, Beevi SS, Jose J, (2020) Annexin A6 modulates TBC1D15/Rab7/StARD3 axis to control endosomal cholesterol export in NPC1 cells. Cell Mol Life Sci 77: 2839–2857. 10.1007/s00018-019-03330-y 31664461 PMC7326902

[bib95] Meng B, Abdullahi A, Ferreira I, Goonawardane N, Saito A, Kimura I, Yamasoba D, Gerber PP, Fatihi S, Rathore S, (2022) Altered TMPRSS2 usage by SARS-CoV-2 Omicron impacts infectivity and fusogenicity. Nature 603: 706–714. 10.1038/s41586-022-04474-x 35104837 PMC8942856

[bib96] Mesmin B, Maxfield FR (2009) Intracellular sterol dynamics. Biochim Biophys Acta 1791: 636–645. 10.1016/j.bbalip.2009.03.002 19286471 PMC2696574

[bib97] Mester P, Amend P, Schmid S, Muller M, Buechler C, Pavel V (2023) Plasma proprotein convertase subtilisin/kexin type 9 (PCSK9) as a possible biomarker for severe COVID-19. Viruses 15: 1511. 10.3390/v15071511 37515197 PMC10385877

[bib98] Musiol A, Gran S, Ehrhardt C, Ludwig S, Grewal T, Gerke V, Rescher U (2013) Annexin A6-balanced late endosomal cholesterol controls influenza A replication and propagation. mBio 4: e00608-13. 10.1128/mBio.00608-13 24194536 PMC3892785

[bib99] Navarese EP, Podhajski P, Gurbel PA, Grzelakowska K, Ruscio E, Tantry U, Magielski P, Kubica A, Niezgoda P, Adamski P, (2023) PCSK9 inhibition during the inflammatory stage of SARS-CoV-2 infection. J Am Coll Cardiol 81: 224–234. 10.1016/j.jacc.2022.10.030 36653090 PMC9842071

[bib100] Nejat R, Torshizi MF, Najafi DJ (2023) S protein, ACE2 and host cell proteases in SARS-CoV-2 cell entry and infectivity; is soluble ACE2 a two blade sword? A narrative review. Vaccines (Basel) 11: 204. 10.3390/vaccines11020204 36851081 PMC9968219

[bib101] Ng WH, Tipih T, Makoah NA, Vermeulen JG, Goedhals D, Sempa JB, Burt FJ, Taylor A, Mahalingam S (2021) Comorbidities in SARS-CoV-2 patients: A systematic review and meta-analysis. mBio 12: e03647-20. 10.1128/mBio.03647-20 33563817 PMC7885108

[bib102] Ortiz ME, Thurman A, Pezzulo AA, Leidinger MR, Klesney-Tait JA, Karp PH, Tan P, Wohlford-Lenane C, McCray PB, Jr, Meyerholz DK (2020) Heterogeneous expression of the SARS-Coronavirus-2 receptor ACE2 in the human respiratory tract. EBioMedicine 60: 102976. 10.1016/j.ebiom.2020.102976 32971472 PMC7505653

[bib103] Ottinger EA, Kao ML, Carrillo-Carrasco N, Yanjanin N, Shankar RK, Janssen M, Brewster M, Scott I, Xu X, Cradock J, (2014) Collaborative development of 2-hydroxypropyl-β-cyclodextrin for the treatment of Niemann-Pick type C1 disease. Curr Top Med Chem 14: 330–339. 10.2174/1568026613666131127160118 24283970 PMC4048128

[bib104] Ou X, Liu Y, Lei X, Li P, Mi D, Ren L, Guo L, Guo R, Chen T, Hu J, (2020) Characterization of spike glycoprotein of SARS-CoV-2 on virus entry and its immune cross-reactivity with SARS-CoV. Nat Commun 11: 1620. 10.1038/s41467-020-15562-9 32221306 PMC7100515

[bib105] Palacios-Rapalo SN, De Jesús-González LA, Cordero-Rivera CD, Farfan-Morales CN, Osuna-Ramos JF, Martinez-Mier G, Quistián-Galván J, Muñoz-Pérez A, Bernal-Dolores V, del Angel RM, (2021) Cholesterol-rich lipid rafts as platforms for SARS-CoV-2 entry. Front Immunol 12: 796855. 10.3389/fimmu.2021.796855 34975904 PMC8719300

[bib106] Parker RA, Garcia R, Ryan CS, Liu X, Shipkova P, Livanov V, Patel P, Ho SP (2013) Bile acid and sterol metabolism with combined HMG-CoA reductase and PCSK9 suppression. J Lipid Res 54: 2400–2409. 10.1194/jlr.M038331 23614904 PMC3735938

[bib107] Pienkos SM, Moore AR, Guan J, Levitt JE, Matthay MA, Baron RM, Conlon J, McAuley DF, O’Kane CM, Rogers AJ (2023) Effect of total cholesterol and statin therapy on mortality in ARDS patients: A secondary analysis of the SAILS and HARP-2 trials. Crit Care 27: 126. 10.1186/s13054-023-04387-9 36978134 PMC10053133

[bib108] Pirillo A, Catapano AL, Norata GD (2015) HDL in infectious diseases and sepsis. Handb Exp Pharmacol 224: 483–508. 10.1007/978-3-319-09665-0_15 25522999

[bib109] Porter FD, Scherrer DE, Lanier MH, Langmade SJ, Molugu V, Gale SE, Olzeski D, Sidhu R, Dietzen DJ, Fu R, (2010) Cholesterol oxidation products are sensitive and specific blood-based biomarkers for Niemann-Pick C1 disease. Sci Transl Med 2: 56ra81. 10.1126/scitranslmed.3001417 PMC3170139 21048217

[bib110] Potere N, Garrad E, Kanthi Y, Di Nisio M, Kaplanski G, Bonaventura A, Connors JM, De Caterina R, Abbate A (2023) NLRP3 inflammasome and interleukin-1 contributions to COVID-19-associated coagulopathy and immunothrombosis. Cardiovasc Res 119: 2046–2060. 10.1093/cvr/cvad084 37253117 PMC10893977

[bib111] Prabhakara C, Godbole R, Sil P, Jahnavi S, Gulzar SE, van Zanten TS, Sheth D, Subhash N, Chandra A, Shivaraj A, (2021) Strategies to target SARS-CoV-2 entry and infection using dual mechanisms of inhibition by acidification inhibitors. PLoS Pathog 17: e1009706. 10.1371/journal.ppat.1009706 34252168 PMC8297935

[bib112] Pramfalk C, Jiang ZY, Parini P (2011) Hepatic niemann-pick C1-like 1. Curr Opin Lipidol 22: 225–230. 10.1097/MOL.0b013e3283468c28 21494140

[bib113] Rajasekharan S, Milan Bonotto R, Nascimento Alves L, Kazungu Y, Poggianella M, Martinez-Orellana P, Skoko N, Polez S, Marcello A (2021) Inhibitors of protein glycosylation are active against the coronavirus severe acute respiratory syndrome coronavirus SARS-CoV-2. Viruses 13: 808. 10.3390/v13050808 33946304 PMC8144969

[bib114] Raut P, Waters H, Zimmberberg J, Obeng B, Gosse J, Hess ST (2022) Localization-Based super-resolution microscopy reveals relationship between SARS-CoV2 spike and phosphatidylinositol (4,5)-bisphosphate. Proc SPIE Int Soc Opt Eng 11965: 1196503. 10.1117/12.2613460 36051945 PMC9432428

[bib115] Redondo-Morata L, Lea Sanford R, Andersen OS, Scheuring S (2016) Effect of statins on the nanomechanical properties of supported lipid bilayers. Biophys J 111: 363–372. 10.1016/j.bpj.2016.06.016 27463138 PMC4968420

[bib116] Reiner Z, Hatamipour M, Banach M, Pirro M, Al-Rasadi K, Jamialahmadi T, Radenkovic D, Montecucco F, Sahebkar A (2020) Statins and the COVID-19 main protease: In silico evidence on direct interaction. Arch Med Sci 16: 490–496. 10.5114/aoms.2020.94655 32399094 PMC7212226

[bib117] Reiss K, Bhakdi S (2017) The plasma membrane: Penultimate regulator of ADAM sheddase function. Biochim Biophys Acta Mol Cell Res 1864: 2082–2087. 10.1016/j.bbamcr.2017.06.006 28624437

[bib118] Reverter M, Rentero C, Garcia-Melero A, Hoque M, Vila de Muga S, Alvarez-Guaita A, Conway JR, Wood P, Cairns R, Lykopoulou L, (2014) Cholesterol regulates Syntaxin 6 trafficking at trans-Golgi network endosomal boundaries. Cell Rep 7: 883–897. 10.1016/j.celrep.2014.03.043 24746815

[bib119] Ripa I, Andreu S, Lopez-Guerrero JA, Bello-Morales R (2021) Membrane rafts: Portals for viral entry. Front Microbiol 12: 631274. 10.3389/fmicb.2021.631274 33613502 PMC7890030

[bib120] Rodriguez-Gil JL, Watkins-Chow DE, Baxter LL, Yokoyama T, Zerfas PM, Starost MF, Gahl WA, Malicdan MCV, Porter FD, Platt FM, (2019) NPC1 deficiency in mice is associated with fetal growth restriction, neonatal lethality and abnormal lung pathology. J Clin Med 9: 12. 10.3390/jcm9010012 31861571 PMC7019814

[bib121] Roszell BR, Tao JQ, Yu KJ, Huang S, Bates SR (2012) Characterization of the Niemann-Pick C pathway in alveolar type II cells and lamellar bodies of the lung. Am J Physiol Lung Cell Mol Physiol 302: L919–L932. 10.1152/ajplung.00383.2011 22367786 PMC3362154

[bib122] Rudajev V, Novotny J (2022) Cholesterol as a key player in amyloid β-mediated toxicity in Alzheimer's disease. Front Mol Neurosci 15: 937056. 10.3389/fnmol.2022.937056 36090253 PMC9453481

[bib123] Ruscica M, Macchi C, Iodice S, Tersalvi G, Rota I, Ghidini S, Terranova L, Valenti L, Amati F, Aliberti S, (2021) Prognostic parameters of in-hospital mortality in COVID-19 patients-An Italian experience. Eur J Clin Invest 51: e13629. 10.1111/eci.13629 34184268 PMC8420178

[bib124] Sanders DW, Jumper CC, Ackerman PJ, Bracha D, Donlic A, Kim H, Kenney D, Castello-Serrano I, Suzuki S, Tamura T, (2021) SARS-CoV-2 requires cholesterol for viral entry and pathological syncytia formation. Elife 10: e65962. 10.7554/eLife.65962 33890572 PMC8104966

[bib125] Sasidhar MV, Chevooru SK, Eickelberg O, Hartung HP, Neuhaus O (2017) Downregulation of monocytic differentiation via modulation of CD147 by 3-hydroxy-3-methylglutaryl coenzyme A reductase inhibitors. PLoS One 12: e0189701. 10.1371/journal.pone.0189701 29253870 PMC5734787

[bib126] Schloer S, Goretzko J, Kuhnl A, Brunotte L, Ludwig S, Rescher U (2019) The clinically licensed antifungal drug itraconazole inhibits influenza virus in vitro and in vivo. Emerg Microbes Infect 8: 80–93. 10.1080/22221751.2018.1559709 30866762 PMC6455256

[bib127] Schloer S, Brunotte L, Goretzko J, Mecate-Zambrano A, Korthals N, Gerke V, Ludwig S, Rescher U (2020a) Targeting the endolysosomal host-SARS-CoV-2 interface by clinically licensed functional inhibitors of acid sphingomyelinase (FIASMA) including the antidepressant fluoxetine. Emerg Microbes Infect 9: 2245–2255. 10.1080/22221751.2020.1829082 32975484 PMC7594754

[bib128] Schloer S, Goretzko J, Pleschka S, Ludwig S, Rescher U (2020b) Combinatory treatment with oseltamivir and itraconazole targeting both virus and host factors in influenza A virus infection. Viruses 12: 703. 10.3390/v12070703 32610711 PMC7412427

[bib129] Schloer S, Brunotte L, Mecate-Zambrano A, Zheng S, Tang J, Ludwig S, Rescher U (2021) Drug synergy of combinatory treatment with remdesivir and the repurposed drugs fluoxetine and itraconazole effectively impairs SARS-CoV-2 infection in vitro. Br J Pharmacol 178: 2339–2350. 10.1111/bph.15418 33825201 PMC8251190

[bib130] Schloer S, Treuherz D, Faist A, de Witt M, Wunderlich K, Wiewrodt R, Wiebe K, Barth P, Walzlein JH, Kummer S, (2022) 3D Ex vivo tissue platforms to investigate the early phases of influenza a virus- and SARS-CoV-2-induced respiratory diseases. Emerg Microbes Infect 11: 2160–2175. 10.1080/22221751.2022.2117101 36000328 PMC9518268

[bib131] Self WH, Semler MW, Leither LM, Casey JD, Angus DC, Brower RG, Chang SY, Collins SP, Eppensteiner JC, Filbin MR, (2020) Effect of hydroxychloroquine on clinical status at 14 days in hospitalized patients with COVID-19: A randomized clinical trial. JAMA 324: 2165–2176. 10.1001/jama.2020.22240 33165621 PMC7653542

[bib132] Shafiee A, Teymouri Athar MM, Mozhgani SH (2023) A twisting tale of misinformation: Should ivermectin be approved as a treatment for COVID-19 disease? Future Virol 18: 137–139. 10.2217/fvl-2023-0006 PMC10005062 36915278

[bib133] Sieurin J, Branden G, Magnusson C, Hergens MP, Kosidou K (2022) A population-based cohort study of sex and risk of severe outcomes in covid-19. Eur J Epidemiol 37: 1159–1169. 10.1007/s10654-022-00919-9 36301399 PMC9607822

[bib134] Silhol F, Sarlon G, Deharo JC, Vaisse B (2020) Downregulation of ACE2 induces overstimulation of the renin-angiotensin system in COVID-19: Should we block the renin-angiotensin system? Hypertens Res 43: 854–856. 10.1038/s41440-020-0476-3 32439915 PMC7242178

[bib135] Smaldone C, Brugaletta S, Pazzano V, Liuzzo G (2009) Immunomodulator activity of 3-hydroxy-3-methilglutaryl-CoA inhibitors. Cardiovasc Hematol Agents Med Chem 7: 279–294. 10.2174/187152509789541864 19663791

[bib136] Stertz S, Reichelt M, Spiegel M, Kuri T, Martinez-Sobrido L, Garcia-Sastre A, Weber F, Kochs G (2007) The intracellular sites of early replication and budding of SARS-coronavirus. Virology 361: 304–315. 10.1016/j.virol.2006.11.027 17210170 PMC7103305

[bib137] Stewart TG, Rebolledo PA, Mourad A, Lindsell CJ, Boulware DR, McCarthy MW, Thicklin F, Garcia Del Sol IT, Bramante CT, Lenert LA, (2023) Higher-dose fluvoxamine and time to sustained recovery in outpatients with COVID-19: The ACTIV-6 randomized clinical trial. JAMA 330: 2354–2363. 10.1001/jama.2023.23363 37976072 PMC10656670

[bib138] Stiekema LCA, Willemsen L, Kaiser Y, Prange KHM, Wareham NJ, Boekholdt SM, Kuijk C, de Winther MPJ, Voermans C, Nahrendorf M, (2021) Impact of cholesterol on proinflammatory monocyte production by the bone marrow. Eur Heart J 42: 4309–4320. 10.1093/eurheartj/ehab465 34343254 PMC8572558

[bib139] Sturley SL, Rajakumar T, Hammond N, Higaki K, Marka Z, Marka S, Munkacsi AB (2020) Potential COVID-19 therapeutics from a rare disease: Weaponizing lipid dysregulation to combat viral infectivity. J Lipid Res 61: 972–982. 10.1194/jlr.R120000851 32457038 PMC7328045

[bib140] Sudhop T, Reber M, Tribble D, Sapre A, Taggart W, Gibbons P, Musliner T, von Bergmann K, Lutjohann D (2009) Changes in cholesterol absorption and cholesterol synthesis caused by ezetimibe and/or simvastatin in men. J Lipid Res 50: 2117–2123. 10.1194/jlr.P900004-JLR200 19380898 PMC2739752

[bib141] Sumbria D, Berber E, Mathayan M, Rouse BT (2020) Virus infections and host metabolism-can we manage the interactions? Front Immunol 11: 594963. 10.3389/fimmu.2020.594963 33613518 PMC7887310

[bib142] Sundararaman SS, Doring Y, van der Vorst EPC (2021) PCSK9: A multi-faceted protein that is involved in cardiovascular biology. Biomedicines 9: 793. 10.3390/biomedicines9070793 34356856 PMC8301306

[bib143] Swanson KV, Deng M, Ting JP (2019) The NLRP3 inflammasome: Molecular activation and regulation to therapeutics. Nat Rev Immunol 19: 477–489. 10.1038/s41577-019-0165-0 31036962 PMC7807242

[bib144] Talukder P, Saha A, Roy S, Ghosh G, Roy DD, Barua S (2023) Drugs for COVID-19 treatment: A new challenge. Appl Biochem Biotechnol 195: 3653–3670. 10.1007/s12010-023-04439-4 36961509 PMC10037400

[bib145] Tang T, Bidon M, Jaimes JA, Whittaker GR, Daniel S (2020) Coronavirus membrane fusion mechanism offers a potential target for antiviral development. Antivir Res 178: 104792. 10.1016/j.antiviral.2020.104792 32272173 PMC7194977

[bib146] Tang T, Jaimes JA, Bidon MK, Straus MR, Daniel S, Whittaker GR (2021a) Proteolytic activation of SARS-CoV-2 spike at the S1/S2 boundary: Potential role of proteases beyond furin. ACS Infect Dis 7: 264–272. 10.1021/acsinfecdis.0c00701 33432808

[bib147] Tang Y, Hu L, Liu Y, Zhou B, Qin X, Ye J, Shen M, Wu Z, Zhang P (2021b) Possible mechanisms of cholesterol elevation aggravating COVID-19. Int J Med Sci 18: 3533–3543. 10.7150/ijms.62021 34522180 PMC8436106

[bib148] Teixeira L, Temerozo JR, Pereira-Dutra FS, Ferreira AC, Mattos M, Goncalves BS, Sacramento CQ, Palhinha L, Cunha-Fernandes T, Dias SSG, (2022) Simvastatin downregulates the SARS-CoV-2-induced inflammatory response and impairs viral infection through disruption of lipid rafts. Front Immunol 13: 820131. 10.3389/fimmu.2022.820131 35251001 PMC8895251

[bib149] Thorp EB, Gallagher TM (2004) Requirements for CEACAMs and cholesterol during murine coronavirus cell entry. J Virol 78: 2682–2692. 10.1128/jvi.78.6.2682-2692.2004 14990688 PMC353758

[bib150] Trinh MN, Lu F, Li X, Das A, Liang Q, De Brabander JK, Brown MS, Goldstein JL (2017) Triazoles inhibit cholesterol export from lysosomes by binding to NPC1. Proc Natl Acad Sci U S A 114: 89–94. 10.1073/pnas.1619571114 27994139 PMC5224357

[bib151] Tudorache IF, Trusca VG, Gafencu AV (2017) Apolipoprotein E - a multifunctional protein with implications in various pathologies as a result of its structural features. Comput Struct Biotechnol J 15: 359–365. 10.1016/j.csbj.2017.05.003 28660014 PMC5476973

[bib152] Usenko T, Miroshnikova V, Bezrukova A, Basharova K, Landa S, Korobova Z, Liubimova N, Vlasov I, Nikolaev M, Izyumchenko A, (2023) Fraction of plasma exomeres and low-density lipoprotein cholesterol as a predictor of fatal outcome of COVID-19. PLoS One 18: e0278083. 10.1371/journal.pone.0278083 36758022 PMC9910704

[bib153] Vainio S, Bykov I, Hermansson M, Jokitalo E, Somerharju P, Ikonen E (2005) Defective insulin receptor activation and altered lipid rafts in Niemann-Pick type C disease hepatocytes. Biochem J 391: 465–472. 10.1042/BJ20050460 15943586 PMC1276947

[bib154] Verma DK, Kapoor S, Das S, Thakur KG (2022) Potential inhibitors of SARS-CoV-2 main protease (M(pro)) identified from the library of FDA-approved drugs using molecular docking studies. Biomedicines 11: 85. 10.3390/biomedicines11010085 36672593 PMC9856154

[bib155] Wang H, Yang P, Liu K, Guo F, Zhang Y, Zhang G, Jiang C (2008) SARS coronavirus entry into host cells through a novel clathrin- and caveolae-independent endocytic pathway. Cell Res 18: 290–301. 10.1038/cr.2008.15 18227861 PMC7091891

[bib156] Wang K, Chen W, Zhang Z, Deng Y, Lian JQ, Du P, Wei D, Zhang Y, Sun XX, Gong L, (2020a) CD147-spike protein is a novel route for SARS-CoV-2 infection to host cells. Signal Transduct Target Ther 5: 283. 10.1038/s41392-020-00426-x 33277466 PMC7714896

[bib157] Wang S, Li W, Hui H, Tiwari SK, Zhang Q, Croker BA, Rawlings S, Smith D, Carlin AF, Rana TM (2020b) Cholesterol 25-Hydroxylase inhibits SARS-CoV-2 and other coronaviruses by depleting membrane cholesterol. EMBO J 39: e106057. 10.15252/embj.2020106057 32944968 PMC7537045

[bib158] Wang G, Deng J, Li J, Wu C, Dong H, Wu S, Zhong Y (2021) The role of high-density lipoprotein in COVID-19. Front Pharmacol 12: 720283. 10.3389/fphar.2021.720283 34335279 PMC8322438

[bib159] Wang T, Cao Y, Zhang H, Wang Z, Man CH, Yang Y, Chen L, Xu S, Yan X, Zheng Q, (2022) COVID-19 metabolism: Mechanisms and therapeutic targets. MedComm 3: e157. 10.1002/mco2.157 35958432 PMC9363584

[bib160] Wang H, Yuan Z, Pavel MA, Jablonski SM, Jablonski J, Hobson R, Valente S, Reddy CB, Hansen SB (2023) The role of high cholesterol in SARS-CoV-2 infectivity. J Biol Chem 299: 104763. 10.1016/j.jbc.2023.104763 37119851 PMC10140059

[bib161] Wen W, Chen C, Tang J, Wang C, Zhou M, Cheng Y, Zhou X, Wu Q, Zhang X, Feng Z, (2022) Efficacy and safety of three new oral antiviral treatment (molnupiravir, fluvoxamine and Paxlovid) for COVID-19. A meta-analysis. Ann Med 54: 516–523. 10.1080/07853890.2022.2034936 35118917 PMC8820829

[bib162] Westheim AJF, Bitorina AV, Theys J, Shiri-Sverdlov R (2021) COVID-19 infection, progression, and vaccination: Focus on obesity and related metabolic disturbances. Obes Rev 22: e13313. 10.1111/obr.13313 34269511 PMC8420274

[bib163] Wing PAC, Schmidt NM, Peters R, Erdmann M, Brown R, Wang H, Swadling L, COVIDsortium Investigators, Newman J, Thakur N, (2023) An ACAT inhibitor suppresses SARS-CoV-2 replication and boosts antiviral T cell activity. PLoS Pathog 19: e1011323. 10.1371/journal.ppat.1011323 37134108 PMC10202285

[bib164] Wu J, Chen L, Qin C, Huo F, Liang X, Yang X, Zhang K, Lin P, Liu J, Feng Z, (2022) CD147 contributes to SARS-CoV-2-induced pulmonary fibrosis. Signal Transduct Target Ther 7: 382. 10.1038/s41392-022-01230-5 36424379 PMC9691700

[bib165] Xie B, Njoroge W, Dowling LM, Sule-Suso J, Cinque G, Yang Y (2022) Detection of lipid efflux from foam cell models using a label-free infrared method. Analyst 147: 5372–5385. 10.1039/d2an01041k 36285592

[bib166] Xiong H, Callaghan D, Jones A, Walker DG, Lue LF, Beach TG, Sue LI, Woulfe J, Xu H, Stanimirovic DB, (2008) Cholesterol retention in Alzheimer's brain is responsible for high beta- and gamma-secretase activities and Abeta production. Neurobiol Dis 29: 422–437. 10.1016/j.nbd.2007.10.005 18086530 PMC2720683

[bib167] Yang N, Shen HM (2020) Targeting the endocytic pathway and autophagy process as a novel therapeutic strategy in COVID-19. Int J Biol Sci 16: 1724–1731. 10.7150/ijbs.45498 32226290 PMC7098027

[bib168] Yuan Z, Hansen SB (2023) Cholesterol regulation of membrane proteins revealed by two-color super-resolution imaging. Membranes (Basel) 13: 250. 10.3390/membranes13020250 36837753 PMC9966874

[bib169] Yuan Z, Pavel MA, Wang H, Kwachukwu JC, Mediouni S, Jablonski JA, Nettles KW, Reddy CB, Valente ST, Hansen SB (2022) Hydroxychloroquine blocks SARS-CoV-2 entry into the endocytic pathway in mammalian cell culture. Commun Biol 5: 958. 10.1038/s42003-022-03841-8 36104427 PMC9472185

[bib170] Zang R, Case JB, Yutuc E, Ma X, Shen S, Gomez Castro MF, Liu Z, Zeng Q, Zhao H, Son J, (2020) Cholesterol 25-hydroxylase suppresses SARS-CoV-2 replication by blocking membrane fusion. Proc Natl Acad Sci U S A 117: 32105–32113. 10.1073/pnas.2012197117 33239446 PMC7749331

[bib171] Zhao M, Luo Z, He H, Shen B, Liang J, Zhang J, Ye J, Xu Y, Wang Z, Ye D, (2021) Decreased low-density lipoprotein cholesterol level indicates poor prognosis of severe and critical COVID-19 patients: A retrospective, single-center study. Front Med (Lausanne) 8: 585851. 10.3389/fmed.2021.585851 34124081 PMC8187559

[bib172] Zhou Y, Vedantham P, Lu K, Agudelo J, Carrion R, Jr, Nunneley JW, Barnard D, Pohlmann S, McKerrow JH, Renslo AR, (2015) Protease inhibitors targeting coronavirus and filovirus entry. Antivir Res 116: 76–84. 10.1016/j.antiviral.2015.01.011 25666761 PMC4774534

[bib173] Zhou N, Pan T, Zhang J, Li Q, Zhang X, Bai C, Huang F, Peng T, Zhang J, Liu C, (2016) Glycopeptide antibiotics potently inhibit cathepsin L in the late endosome/lysosome and block the entry of Ebola virus, Middle East respiratory syndrome coronavirus (MERS-CoV), and severe acute respiratory syndrome coronavirus (SARS-CoV). J Biol Chem 291: 9218–9232. 10.1074/jbc.M116.716100 26953343 PMC4861487

[bib174] Zivkovic S, Maric G, Cvetinovic N, Lepojevic-Stefanovic D, Bozic Cvijan B (2023) Anti-inflammatory effects of lipid-lowering drugs and supplements-A narrative review. Nutrients 15: 1517. 10.3390/nu15061517 36986246 PMC10053759

